# HSDL2 knockdown promotes the progression of cholangiocarcinoma by inhibiting ferroptosis through the P53/SLC7A11 axis

**DOI:** 10.1186/s12957-023-03176-6

**Published:** 2023-09-18

**Authors:** Shuoshuo Ma, Yang Ma, Feiyu Qi, Jiasheng Lei, Fangfang Chen, Wanliang Sun, Dongdong Wang, Shuo Zhou, Zhong Liu, Zheng Lu, Dengyong Zhang

**Affiliations:** 1 Department of General Surgery, The First Affiliated Hospital of Bengbu Medical College, NO. 287, Changhuai Road, Longzihu district, Bengbu, 233000 Anhui China; 2 Liver Transplantation Center and Hepatobiliary and Pancreatic Surgery, Sichuan Cancer Hospital and Institute, Sichuan Cancer Center, School of Medicine, University of Electronic Science and Technology of China, Chengdu, China; 3 Department of Translational Molecular Pathology, The University of Texas MD Anderson Cancer Center, Houston, 77030 USA

**Keywords:** HSDL2, Cholangiocarcinoma, Proliferation, Ferroptosis, Prognosis

## Abstract

**Background:**

Human hydroxysteroid dehydrogenase-like 2 (HSDL2), which regulates cancer progression, is involved in lipid metabolism. However, the role of HSDL2 in cholangiocarcinoma (CCA) and the mechanism by which it regulates CCA progression by modulating ferroptosis are unclear.

**Methods:**

HSDL2 expression levels in CCA cells and tissues were determined by quantitative real-time polymerase chain reaction (qRT-PCR), western blotting, and immunohistochemistry. The overall survival and disease-free survival of patients with high vs. low HSDL2 expression were evaluated using Kaplan-Meier curves. The proliferation, migration, and invasion of CCA cells were assessed using Cell Counting Kit-8, colony formation, 5-ethynyl-2′-deoxyuridine DNA synthesis, and transwell assays. The effect of p53 on tumor growth was explored using a xenograft mouse model. The expression of SLC7A11 in patients with CCA was analyzed using immunofluorescence. Ferroptosis levels were measured by flow cytometry, malondialdehyde assay, and glutathione assay. HSDL2-regulated signaling pathways were analyzed by transcriptome sequencing. The correlation between p53 and SLC7A11 was assessed using bioinformatics and luciferase reporter assays.

**Results:**

HSDL2 expression was lower in primary human CCA tissues than in matched adjacent non-tumorous bile duct tissues. HSDL2 downregulation was a significant risk factor for shorter overall survival and disease-free survival in patients with CCA. In addition, HSDL2 knockdown enhanced the proliferation, migration, and invasion of CCA cells. The transcriptome analysis of HSDL2 knockdown cells showed that differentially expressed genes were significantly enriched in the p53 signaling pathway, and HSDL2 downregulation increased SLC7A11 levels. These findings were consistent with the qRT-PCR and western blotting results. Other experiments showed that p53 expression modulated the effect of HSDL2 on CCA proliferation in vivo and in vitro and that p53 bound to the SLC7A11 promoter to inhibit ferroptosis.

**Conclusions:**

HSDL2 knockdown promotes CCA progression by inhibiting ferroptosis through the p53/SLC7A11 axis. Thus, HSDL2 is a potential prognostic marker and therapeutic target for CCA.

**Supplementary Information:**

The online version contains supplementary material available at 10.1186/s12957-023-03176-6.

## Introduction

Cholangiocarcinoma (CCA), originating from the biliary epithelium, is the second most common primary liver cancer worldwide after hepatocellular carcinoma [1, 2]. Generally, changes in existing serum biomarkers can only be detected in late-stage CCA [3]. Although there have been new advances in the study of the pathogenesis [4] and related treatments of cholangiocarcinoma, including chemotherapy and immunotherapy [5–7], the prognosis of most patients has not been significantly improved. Thus, there is a pressing need to identify effective diagnostic and prognostic biomarkers for early-stage CCA.

HSDL2, composed of an N-terminal catalytic domain and a C-terminal SCP-2 domain, belongs to the short-chain dehydrogenase/reductase (SDR) superfamily of oxidoreductases [8], which catalyze the oxidation and reduction of several substrates, including steroids, sugars, fatty acids, vitamins, and retinoids [9]. HSDL2 expression is dysregulated in various cancers and is correlated with tumor progression [10–14]. HSDL2 is localized to peroxisomes and affects tumor progression by modulating lipid metabolism [15, 16].

Ferroptosis is an iron-dependent type of cell death characterized by increased lipid peroxidation; this type of cell death is distinct from necrosis, apoptosis, and autophagy [17, 18]. Ferroptosis has potential roles in neurogenesis, cancer, and organ dysfunction [18, 19] and is regulated by many genes and signaling pathways associated with tumor processes [20, 21]. Moreover, ferroptosis improves the efficacy of anti-cancer therapies [22, 23].

The tumor suppressor protein p53 is a major barrier against the onset and progression of cancer. From a biochemistry standpoint, p53 mainly acts as a sequence-specific transcription factor that can bind to DNA sequences defined within the genome and affect gene transcription. The p53 molecule plays an important role in many types of cancer, including colorectal cancer [24], prostate cancer [25], lung cancer [26], kidney cancer [27] and other cancers [28]. With the deepening of the research on CCA, the role of p53 in the development of CCA is gradually gaining prominence [28, 29].

However, the correlations among HSDL2, p53 and ferroptosis in CCA cells have not been determined. In this study, we investigated the role of HSDL2 in CCA and the underlying mechanisms through in vitro and in vivo experiments.

## Materials and methods

### Patients and clinical samples

This study retrospectively analyzed 63 patients with CCA admitted to the First Affiliated Hospital of Bengbu Medical College, Anhui Province, China, between 2015 and 2020. All cases were primary patients not undergoing preoperative treatment. Tissue specimens were examined by a pathologist to identify the presence of tumorous and surrounding non-tumorous tissues. Patient characteristics are shown in Table 1. The study was approved by the Research Ethics Committee of Bengbu Medical College (Protocol No. 2021230).

**Table 1:** Association of HSDL2 expression levels with different clinicopathologic characteristics in cholangiocarcinoma

Clinicopathologic characteristics	HSDL2 expression		*p* value
	Low	High	
	Count(n%)	Count(n%)	
Sex			
Male	23(36.5)	10(15.9)	
Female	16(25.4)	14(22.2)	0.182
Age, years			
≤60	15(23.8)	10(15.9)	
>60	24(38.1)	14(22.2)	0.801
Position			
Intrahepatic	3(4.8)	2(3.2)	
Perihilar	20(31.7)	15(23.8)	
Distal	16(25.4)	7(11.1)	0.632
Histologic grade			
Well	1(1.6)	2(3.2)	
Moderate	31(49.2)	14(22.2)	
Poor	7(11.1)	8(12.7)	0.179
Diameter of tumor			
≤3 cm	21(33.3)	20(31.7)	
>3 cm	18(28.6)	4(6.3)	0.017
Number of tumor			
Single	25(39.7)	21(33.3)	0.042
Multiple	14(22.2)	3(4.8)	
Lymphatic metastasis			
Positive	9 (14.3)	4(6.3)	
Negative	23(36.5)	15(23.8)	0.820
Unknown	7(11.1)	5(7.9)	
Distant metastasis			
Positive	1(1.6)	1(1.6)	
Negative	30(47.6)	18(28.6)	1.000
Unknown	8(12.7)	5(7.9)	
Vascular invasion			
Positive	19(30.2)	4(6.3)	
Negative	12(19.0)	11(17.5)	0.028
Unknown	8(12.7)	9(14.3)	
TNM stage			
Stage I-II	19(30.2)	14(22.2)	
Stage III-IV	15(23.8)	8(12.7)	0.565
Unknown	5(7.9)	2(3.2)	
HBV infection			
Positive	4(6.3)	0(0.0)	
Negative	34(54.0)	24(38.1)	0.266
Unknown	1(1.6)	0(0.0)	
ALT			
≤60	10(15.9)	4(6.3)	
>60	28(44.4)	20(31.7)	0.376
Untested	1(1.6)	0(0.0)	
AST			
≤45	8(12.7)	4(6.3)	
>45	30(47.6)	20(31.7)	0.924
Untested	1(1.6)	0(0.0)	
Albumin:globulin			
<1.2	6(9.5)	7(11.1)	
≥1.2	32(50.8)	17(27.0)	0.208
Untested	1(1.6)	0(0.0)	
Cholesterol			
≤ 5.23	18(28.6)	3(4.8)	
>5.23	19(30.2)	20(31.7)	0.005
Untested	2(3.2)	1(1.6)	
Triglyceride			
≤1.7	15(23.8)	5(7.9)	
>1.7	22(34.9)	18(28.6)	0.133
untested	2(3.2)	1(1.6)	
CEA level, ng/mL			
≤5	25(39.7)	17(27.0)	
>5	5(7.9)	4(6.3)	1.000
Untested	9(14.3)	3(4.8)	
AFP level, ng/mL			
≤20	29(46.0)	21(33.3)	
>20	0(0.0)	0(0.0)	—
untested	10(15.9)	3(4.8)	
CA153 level, IU/mL			
≤28	29(46.0)	20(31.7)	
>28	0(0.0)	1(1.6)	0.420
untested	9(14.3)	4(6.3)	
CA199 level, IU/mL			
≤37	9(14.3)	2(3.2)	
>37	21(33.3)	20(31.7)	0.139
Untested	9(14.3)	2(3.2)	

### Cell culture and transfection

Human intrahepatic biliary epithelial cells (HIBEpiCs) and human CCA cells (RBE and HCCC-9810) were grown in Roswell Park Memorial Institute 1640 medium (Gibco, USA, 22400089) with 10% fetal bovine serum (Gibco, USA, 16140071) and antibiotics (100 µg/mL penicillin and 100 U/mL streptomycin). Cells were incubated in a humidified incubator with 5% CO2 at 37℃. The cells infected with lentiviruses containing HSDL2 overexpression or knockdown plasmids were cultured with puromycin (0.5–5 µg/mL) to select stable cell lines. Plasmids or siRNAs were transiently transfected into CCA cells using Lipofectamine 2000.

### Antibodies, reagents, and constructs

The antibodies used in western blotting, immunofluorescence (IF), and immunohistochemistry (IHC) are shown in Supplementary Table 1. Reagents are shown in Supplementary Table 2.

LV-ShHSDL2 (lentiviral vector control containing luciferase and an HSDL2 knockdown plasmid) and LV-HSDL2 (lentiviral vector control containing luciferase and an HSDL2-expressing plasmid) were processed by GenePharma (Shanghai, China). The cell lines were infected with the lentivirus-based shRNA vector GV344 (hU6-MCS-Ubiquitin-firefly Luciferase-IRES-puromycin) from Genechem (Shanghai, China). Short-interfering RNAs (siRNAs) against p53 (si-p53), a scramble siRNA (siRNA control), a p53 overexpression plasmid, and a negative control plasmid were obtained from GenePharma. Luciferase reporter constructs were designed by inserting SLC7A11 promoter sequences harboring p53-binding sites into the pGL3-basic firefly luciferase vector (GenePharma, Shanghai, China). The sequences of lentiviral plasmids and siRNAs/shRNAs are listed in Supplementary Tables 3 and 4.

### Western blotting

Proteins were extracted using cell lysis buffer (Biosharp, China, BL509A) supplemented with phenylmethylsulfonyl fluoride (Biosharp, China, BL507A). The protein concentration was measured using the bicinchoninic acid protein assay kit (Biosharp, China, BL521A). Proteins were separated by sodium dodecyl sulfate-polyacrylamide gel electrophoresis and transferred to a polyvinylidene fluoride membrane. The membrane was blocked with 5% nonfat milk and incubated with primary antibodies overnight at 4°C and then with secondary antibodies for 1 h at room temperature. The membrane was developed using the Fusion FX SPECTRA Multifunctional Imaging system (Vilber, French).

### Quantitative real-time polymerase chain reaction (qRT-PCR)

Total RNA was obtained using TRIzol reagent. The isolated RNA (1 µg) was reverse-transcribed to complementary DNA (cDNA) using the RevertAid First Strand cDNA Synthesis Kit (Thermo, USA, K1622). qRT-PCR was performed using TB Green® Premix Ex Taq™ II (Tli RNaseH Plus) (Takara Bio, Japan, RR820A) on a StepOnePlus Real-Time PCR System (Applied Biosystem, USA, Cat#4376600). The primers used in qRT-PCR are shown in Supplementary Table 5.

### Cell Counting Kit-8 (CCK-8) assay

Cell viability was assessed using the CCK-8 assay (Biosharp, China, BS350A). Briefly, cells were seeded in 96-well plates (1 × 10^3^ cells per well) and grown for 24 h in a incubator at 37℃ and 5% CO2 using RPMI 1640 medium with 10% fetal bovine serum. Then, the cells were incubated with the CCK-8 solution for 2 h (final concentration: 10%). The absorbance at 450 nm was measured using a Synergy HT Multi-Mode Microplate Reader (BioTek, USA) every 12 h for 2 days.

### Colony formation assay

Cells were seeded in 6-well plates (2 × 10^3^ cells per well) and grown for approximately 2 weeks to allow the development of visible colonies. After that, cells were fixed in 4% paraformaldehyde (Biosharp, China, BL539A) and stained with 0.1% crystal violet (Beyotime, China, C0121-100 mL). Colonies were imaged using a mobile phone camera and quantified using ImageJ version 1.35k (National Institutes of Health, Bethesda, MD, USA).

### Transwell assay

Cell migration and invasion abilities were assayed with a transwell system containing 8.0-µm pores (Corning, USA, CLS3422). Cells in the logarithmic growth phase were synchronized by serum starvation overnight. For cell invasion assays, transwell inserts were coated with 50 µl of a mixture of serum-free RPMI 1640 and Matrigel (1:8). Matrigel was allowed to solidify at 37℃ for 4 h. Synchronized cells in 100 µl serum-free RPMI 1640 were seeded on the upper chamber and allowed to settle for 20 min, and 600 µl of complete RPMI 1640 medium was added to the lower chamber. For the cell migration assay, synchronized cells were seeded on the upper chamber. Cells were incubated in a humidified incubator with 5% CO2 for 24 h at 37℃. The cells that migrated to the lower chamber were washed with PBS three times, fixed with 4% paraformaldehyde for 15 min, and stained with 0.1% crystal violet at room temperature for 30 min. The number of cells that migrated through the pores or invaded the Matrigel was counted. Cells in five representative fields were counted in each membrane.

### IHC and IF

Paraffin sections (3 µm in thickness) of formalin-fixed CCA tissues and adjacent non-tumorous tissues were deparaffinized and rehydrated. Endogenous peroxidase activity was blocked with 3% hydrogen peroxide. The slides were incubated with a primary antibody against HSDL2 (1:200) overnight and then incubated with horseradish peroxidase (HRP)-conjugated secondary antibodies for 1 h at room temperature. Immunohistochemical staining was performed using a Polink-2 Plus polymerized HRP broad DAB-detection system (Zhongshan Biotechnology). The intensity and area of staining were evaluated using the German semi-quantitative scoring method (no staining/not detected = 0; weak staining/light yellow = 1; moderate staining/yellowish brown = 2; and strong staining/brown =3), and the fraction of stained cells was scored in accordance with the following criteria: 0% = 0, 1-24% = 1, 25-49% = 2, 50-74% = 3, and 75-100% = 4. The samples were imaged on an optical microscope (CX23, Olympus, Tokyo, Japan).

IF staining detects the expression of proteins. The tissue sections were incubated with an anti-SLC7A11 antibody (1:200) overnight at 4℃ and an anti-rabbit CoraLite594-conjugated fluorescent secondary antibody for 1 h at room temperature in the dark and stained with 4’,6-diamidino-2-phenylindole (DAPI) for 30 minutes at room temperature in the dark. Protein expression was analyzed using CaseViewer software (3DHISTECH, Budapest, Hungary).

To measure p53 expression, cells were seeded in 6-well plates and cultured for 24 h a incubator at 37℃ and 5% CO2 using RPMI 1640 medium with 10% fetal bovine serum. Cells were fixed in 4% paraformaldehyde, permeabilized in 0.5% Triton X-100, and blocked with 5% bovine serum albumin. Samples were incubated with primary antibodies overnight and then with CoraLite594-conjugated fluorescent secondary antibodies for 1 h at room temperature. Cells were stained with DAPI and imaged on an inverted fluorescence microscope (Observer Z1, Zeiss, Germany).

### Bioinformatics analysis

The differential expression of SLC7A11 between CCA tissues and adjacent healthy bile duct tissues and the relationship of SLC7A11 expression with HSDL2 expression were analyzed using the Gene Expression Profiling Interactive Analysis (GEPIA) website (http://gepia.cancer-pku.cn/). Transcriptome data were analyzed using the BGI platform (https://biosys.bgi.com).

### EdU DNA synthesis assays

Cells were seeded in 6-well plates (2.0 × 104 per well) and grown for 24 h using RPMI 1640 medium with 10% fetal bovine serum. The culture medium was aspirated, and cells were incubated in a medium containing 10 µM EdU solution for 2 h. The cells were fixed in 4% paraformaldehyde, permeabilized in 0.5% Triton X-100, and processed using the BeyoClick EdU Cell Proliferation Kit (Beyotime Biotechnology, Shanghai, China). The samples were imaged on an inverted fluorescence microscope (Observer Z1, ZEISS, Germany).

### Lipid peroxidation assay

Reactive oxygen species (ROS) levels were measured using a superoxide anionic fluorescent probe (Invitrogen, USA, D3861). Cells were seeded in 6-well plates and cultured for 24 h using RPMI 1640 medium with 10% fetal bovine serum. The culture medium was aspirated, and cells were incubated in medium containing 5 µM BODIPY^TM^ 581/591 C11 for 30 min. Cells were harvested, resuspended in PBS, and analyzed using a flow cytometer equipped with a 488-nm argon excitation laser. Fluorescence was measured in the FL1 channel.

### Quantification of glutathione (GSH) and malondialdehyde (MDA) concentrations

GSH concentration was quantified using a commercial kit (Nanjing Jiancheng Biotechnology, China, A006-2-1). MDA concentration was quantified using the lipid peroxidation MDA assay kit (Beyotime, China, S0131).

### Dual-luciferase reporter assay

RBE cells were seeded in 6-well plates until they reached 80% confluence and were then co-transfected with the pGL3-basic-Luc reporter and the internal control plasmid pRL-TK. Then, cells were co-transfected with a p53 overexpression plasmid or an empty vector for 48 h, and luciferase activity was measured using a dual-luciferase assay system (GenePharma, Shanghai, China).

### Xenograft mouse model

For in vivo experiments, 1 × 10^7^ RBE cells (vector + siRNA control, HSDL2 + siRNA control, and HSDL2 + si-p53 cells) were injected into the flank of 4-week-old female BALB/c-nu athymic nude mice (Hangzhou Ziyuan Laboratory Animal Technology Co. Ltd., China; *n* = 5 mice per group). Subcutaneous tumors appeared on day 3 post-injection in the groups injected with vector + siRNA control and HSDL2 + si-p53 cells and on day 5 post-injection in the group injected with HSDL2 + siRNA control cells. In the group injected with vector + siRNA control cells, the model could not be established in one mouse. In the group injected with HSDL2 + si-p53 cells, one mouse died on day 23 post-injection. On day 30 post-injection, mice were euthanized, and tumors were harvested and measured (tumor volume = length × width2 × 0.5). The study protocols were approved by the Research Ethics Committee of Bengbu Medical College (Protocol No. 2021298).

### Statistical analysis

The means of data from two groups were compared using Student’s t-test. The means of data from three or more groups were compared using one-way analysis of variance. The relationship between gene expression and patient characteristics was investigated using the chi-squared test. Survival curves were compared using the log-rank test. All statistical analyses were performed using SPSS version 22.0 (SPSS Inc., Chicago, USA).

## Results

### HSDL2 downregulation in human CCA is associated with poor outcomes

**Fig. 1:**
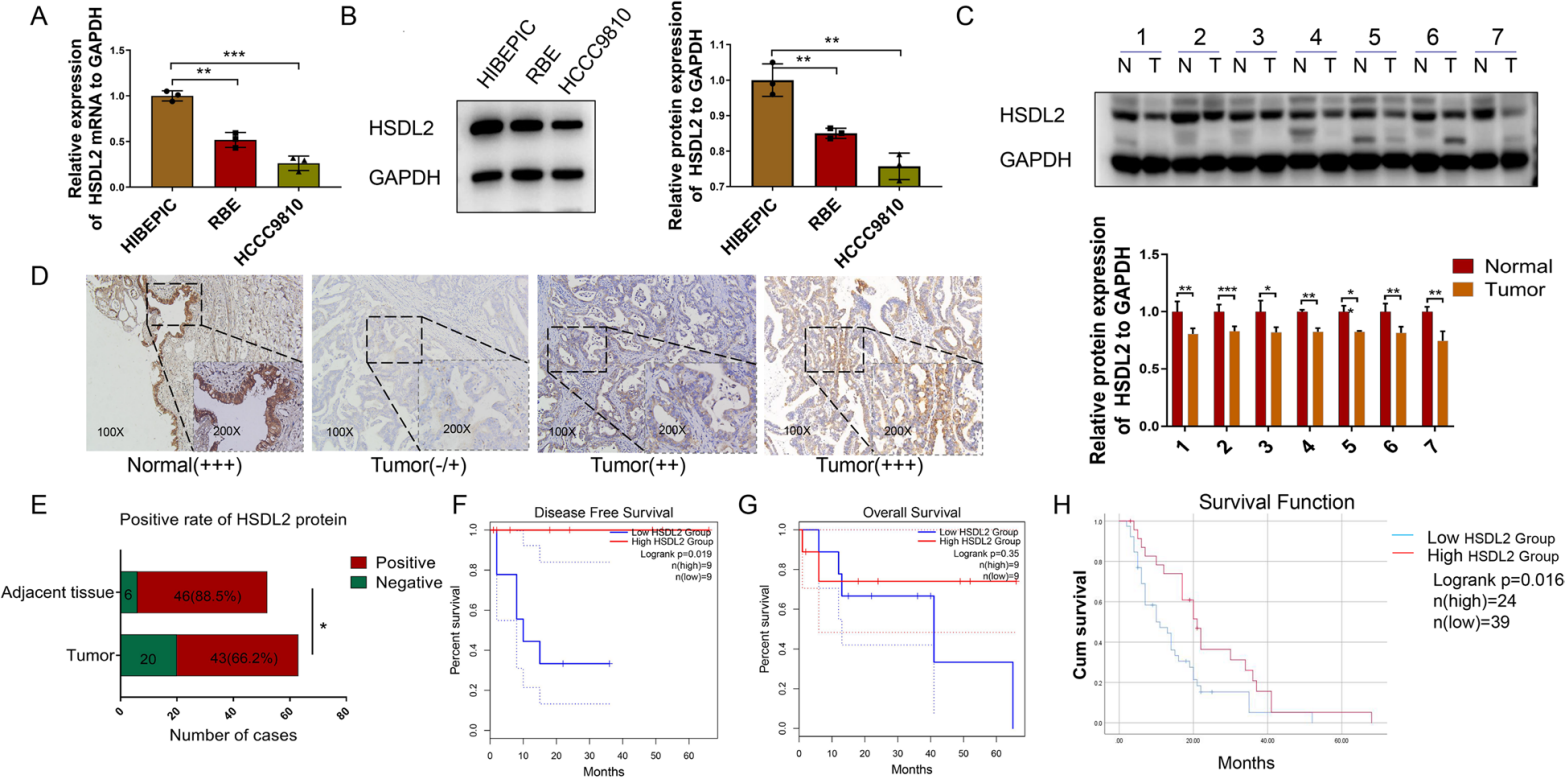
Low HSDL2 expression is related to poor outcomes in patients with cholangiocarcinoma (CCA). **A** HSDL2 mRNA levels in human intrahepatic biliary epithelial cells (HIBEpiCs) and CCA cells (RBE and HCCC9810 cells). **B** HSDL2 protein levels in HIBEpiCs, RBE cells, and HCCC9810 cells. **C** Western blotting of HSDL2 expression in 7 pairs of randomly selected CCA and adjacent non-tumorous tissues. **D** Immunohistochemical analysis of HSDL2 expression in representative CCA and healthy bile duct tissue samples. **E** Positive staining rate of HSDL2 in 63 cases of tumor tissues and 52 cases of adjacent tissues. **F**, **G** Kaplan-Meier curves were applied to analyze differences in DFS (**F**) and OS (**G**) between patients with CCA with different HSDL2 expression levels in terms of OS and DFS from GEPIA database. **H** The plot of HSDL2 expression and prognosis of patients with CCA based on immunohistochemical and clinical data. Data are presented as mean ± SD (*n=*3). *p < 0.05, **p < 0.01, and ***p < 0.001

We previously analyzed CCA microarray datasets from the Gene Expression Omnibus database (three datasets with a total of 353 samples) and The Cancer Genome Atlas (clinical samples of 45 patients) and found that HSDL2 expression was markedly lower in CCA tissues than in matched peritumoral tissues [10]. qRT-PCR (Fig. 1A) and western blotting (Fig. 1B) revealed that HSDL2 levels in two CCA cell lines (RBE and HCCC9810) were significantly lower than those in HIBEpiCs. The expression levels of HSDL2 in seven human CCA samples and matched adjacent healthy bile duct tissues were measured. The results showed that HSDL2 expression was lower in CCA samples than in normal bile duct samples (Fig. 1C). The results of western blotting were in line with the results of IHC (Fig. 1D, E). The relationship between the expression level of HSDL2 and the clinical characteristics of patients with CCA was analyzed (Table 1). HSDL2 expression level was not significantly correlated with age, sex, histological grade, tumor location, lymphatic metastasis, distant metastasis, TNM stage, hepatitis B virus infection, tumor index, or liver function index in patients with CCA. HSDL2 was markedly downregulated in CCA patients with a high number of tumors, large tumor size, vascular invasion, and low total cholesterol levels compared to CCA patients with other features. The log-rank test of two independent cohort datasets from the GEPIA database indicated that CCA patients with low HSDL2 expression had a shorter disease-free survival time than those with high expression (Fig. 1F), but the difference in overall survival was not statistically significant (Fig. 1G). However, HSDL2 downregulation predicted shorter overall survival according to our study of clinical specimens (Fig. 1H). These findings demonstrate that HSDL2 is downregulated in CCA tissues and that HSDL2 downregulation is associated with a poor prognosis.

### HSDL2 knockdown promotes the proliferation, migration, and invasion of CCA cells

**Fig. 2:**
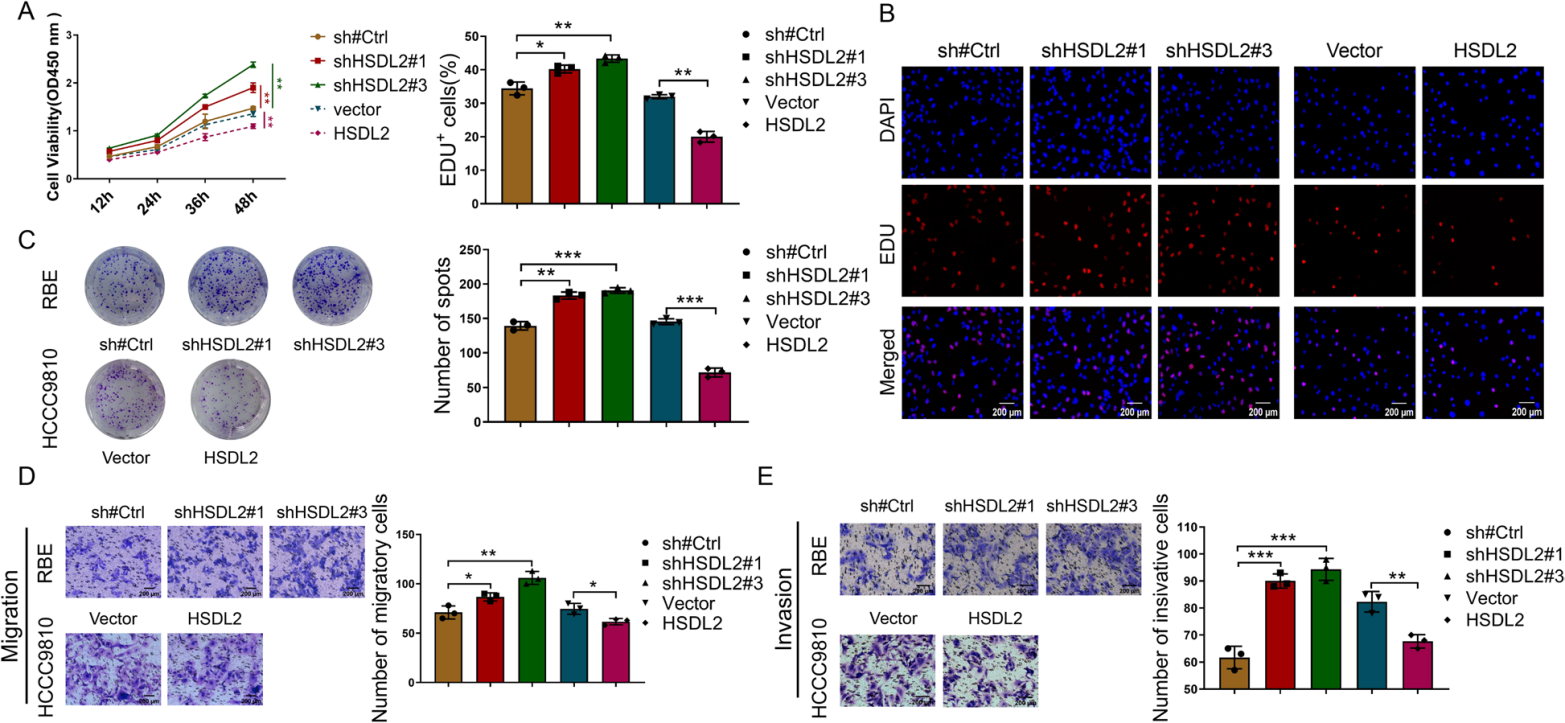
HSDL2 knockdown enhances the proliferation, migration, and invasion of cholangiocarcinoma (CCA) cells. **A**–**C** The proliferation of RBE-sh#Ctrl, RBE-shHSDL2#1, RBE-shHSDL2#3, HCCC9810-Vector, and HCCC9810-HSDL2 cells was assessed by Cell Counting Kit-8 (**A**), 5-ethynyl-2′-deoxyuridine DNA synthesis (**B**), and colony formation (**C**) assays. **D** The migration of RBE-sh#Ctrl, RBE-shHSDL2#1, RBE-shHSDL2#3, HCCC9810-Vector, and HCCC9810-HSDL2 cells was evaluated using the Transwell migration assays. **E** The invasion of RBE-sh#Ctrl, RBE-shHSDL2#1, RBE-shHSDL2#3, HCCC9810-Vector, and HCCC9810-HSDL2 was assessed using Matrigel invasion assays. Data are presented as mean ± SD (*n=*3). *p < 0.05, **p < 0.01, and ***p < 0.001

Three HSDL2-specific short hairpin RNAs (shRNAs) were incorporated into lentiviral vectors, which were transfected into RBE cells to knockdown endogenous HSDL2. The analysis of green fluorescent protein fluorescence intensity indicated that the virus infected the cells with high efficiency (Supplementary Figure 1A). The transfection efficiency for each construct was verified by qRT-PCR (Supplementary Figure 1C) and western blotting (Supplementary Figure 1E). HCCC9810 cells stably expressing HSDL2 were constructed using a similar protocol (Supplementary Figure 1B, D, and F). Proliferation, migration, and invasion were markedly promoted in HSDL2 knockdown cells vs. negative control cells (Fig. 2A–E). In line with these findings, transduction with an HSDL2 overexpression construct (LV-HSDL2) suppressed the proliferation, migration, and invasion of HCCC9810 cells (Fig. 2A–E). These results indicate that HSDL2 knockdown promoted cell proliferation, migration, and invasion, whereas HSDL2 overexpression exerted the opposite effect.

### HSDL2 knockdown promotes the proliferation, migration, and invasion of CCA cells by inhibiting the p53 pathway

**Fig. 3:**
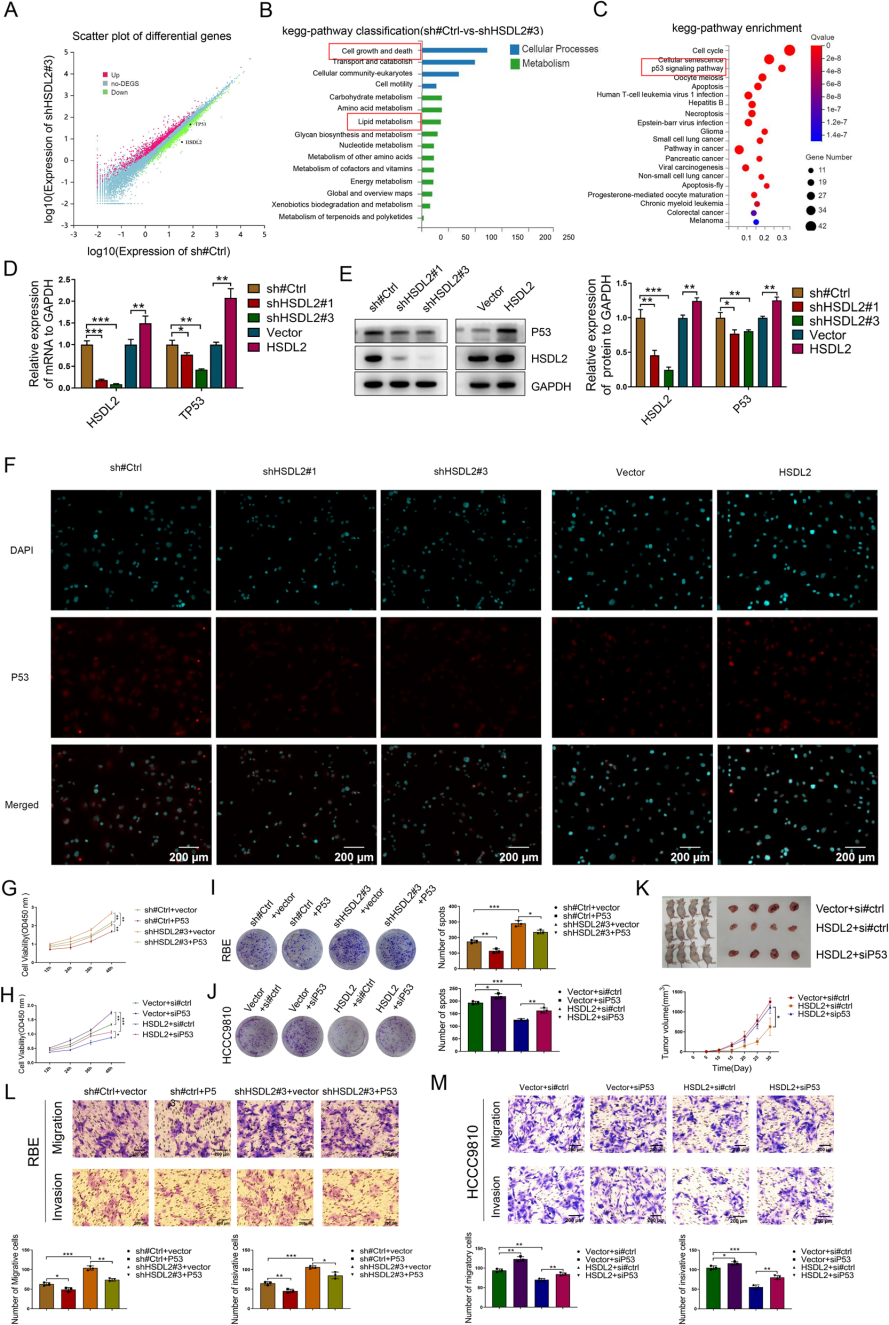
HSDL2 downregulation promotes the proliferation, migration, and invasion of cholangiocarcinoma (CCA) cells by inhibiting the P53 pathway. **A** Scatter plot of differentially expressed genes determined using transcriptome sequencing. **B** KEGG classification and enrichment analysis was performed with differentially expressed genes. **C** KEGG enrichment analysis was performed on differentially expressed genes enriched in the “cell growth and death” gene set. **D**, **E** RBE-sh#Ctrl, RBE-shHSDL2#1, RBE-shHSDL2#3, HCCC9810-Vector, and HCCC9810-HSDL2 cells were subjected to qRT-PCR (**D**) and western blotting with indicated antibodies (**E**). **F** The expression of P53 was analyzed using immunofluorescence staining. **G-J** RBE-sh#Ctrl and RBE-shHSDL2#3 cells transfected with vector or P53 plasmids (**G**, **I**), as well as HCCC9810-Vector and HCCC9810-HSDL2 cells transfected with si#ctrl or siP53 (**H**, **J**), were subjected to cell proliferation assay. **K** Image of xenograft tumors. **L**–**M** RBE-sh#Ctrl and RBE-shHSDL2#3 cells transfected with vector or P53 plasmids (**L**), as well as HCCC9810-Vector and HCCC9810-HSDL2 cells transfected with si#ctrl or siP53 siRNAs (**M**), were subjected to invasion and migration assays. Data are presented as mean ± SD (*n=*3). *p < 0.05, **p < 0.01, and ***p < 0.001

Transcriptome sequencing of HSDL2 knockdown RBE cells was performed. The expression of p53 was lower in these cells than in control cells (Fig. 3A). Kyoto Encyclopedia of Genes and Genomes (KEGG) pathway enrichment analysis showed that differentially expressed genes (DEGs) were significantly enriched in pathways related to cell growth and death and lipid metabolism (Fig. 3B); 122 DEGs enriched in cell growth and death were subjected to further KEGG enrichment analysis. HSDL2 was significantly correlated with the p53 signaling pathway in CCA cells (Fig. 3C). In addition, HSDL2 knockdown decreased the mRNA and protein expression of p53 in RBE cells (Fig. 3D, E). Conversely, HSDL2 overexpression had the opposite effect in HCCC9810 cells (Fig. 3D, E). These results were consistent with those of IF analysis (Fig. 3F).

We explored the effect of p53 expression on the proliferation, migration, and invasion of CCA cells (Supplementary Figure 2A and B). The CCK-8 assay (Fig. 3G) and colony formation assay (Fig. 3I) showed that p53 overexpression suppressed cell proliferation induced by HSDL2 knockdown. Furthermore, it also suppressed the migration and invasion of HSDL2 knockdown REB cells (Fig. 3L). However, p53 downregulation abolished the inhibitory effect of HSDL2 overexpression on the proliferation (Fig. 3H, J, and K), migration and invasion (Fig. 3M) of HCCC9810 cells. These results demonstrated that p53 reversed the effect of HSDL2 in CCA.

### HSDL2 knockdown upregulates SLC7A11 and suppresses ferroptosis in CCA cells

**Fig. 4:**
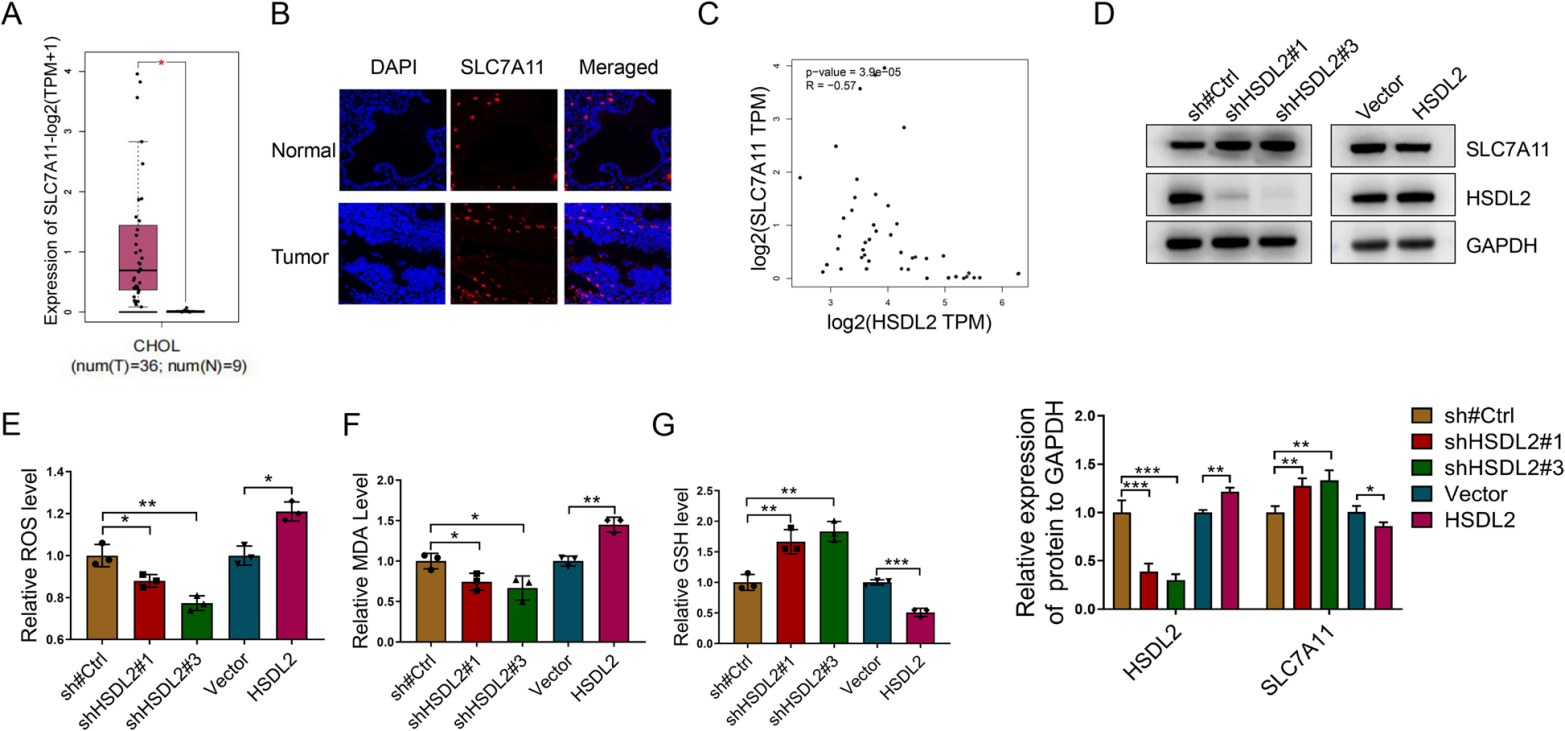
HSDL2 knockdown upregulates SLC7A11 and suppresses ferroptosis in cholangiocarcinoma (CCA) cells. **A** Differential SLC7A11 mRNA expression between CCA tissues and adjacent tissues were determined using the GEPIA database. **B** Immunofluorescence was applied to analyze SLC7A11 expression in CCA tissues and adjacent tissues. **C** Correlation between HSDL2 expression and SLC7A11 expression in CCA datasets from the GEPIA database. **D** Western blotting of HSDL2 and SLC7A11 expression levels in CCA cells. **E**–**G** The relative reactive oxygen species (ROS) (**E**), malonaldehyde (MDA) (**F**), and GSH (**G**) levels in the indicated groups were measured using the corresponding kits. Data are presented as mean ± SD (*n=*3). *p < 0.05, **p < 0.01, and ***p < 0.001

Transcriptome sequencing revealed that HSDL2 knockdown increased the expression of SLC7A11 (log2 fold-change (SH/NC) = 0.65, *p* = 0.11). According to GEPIA, the SLC7A11 level in the CCA tissues was higher than that in healthy bile duct tissues (Fig. 4A). This finding was consistent with the results of IF (Fig. 4B). Additionally, the analysis of GEPIA datasets suggested that HSDL2 expression was negatively correlated with SLC7A11 expression (Fig. 4C). Western blotting showed that SLC7A11 levels were increased in HSDL2 knockdown cells and decreased in HSDL2-overexpressing cells (Fig. 4D). HSDL2 knockdown by shRNA decreased reactive oxygen species (ROS) levels measured using a superoxide anionic fluorescent probe (Fig. 4E). Moreover, HSDL2 knockdown decreased MDA concentration (Fig. 4F) and increased the levels of the antioxidant GSH (Fig. 4G). HSDL2 overexpression had the opposite effect (Fig. 4E–G). These results suggest that HSDL2 downregulation activates SLC7A11 in cells, decreasing lipid ROS levels and inhibiting ferroptosis.

### HSDL2 knockdown suppresses ferroptosis by inhibiting the p53-SLC7A11 pathway

**Fig. 5:**
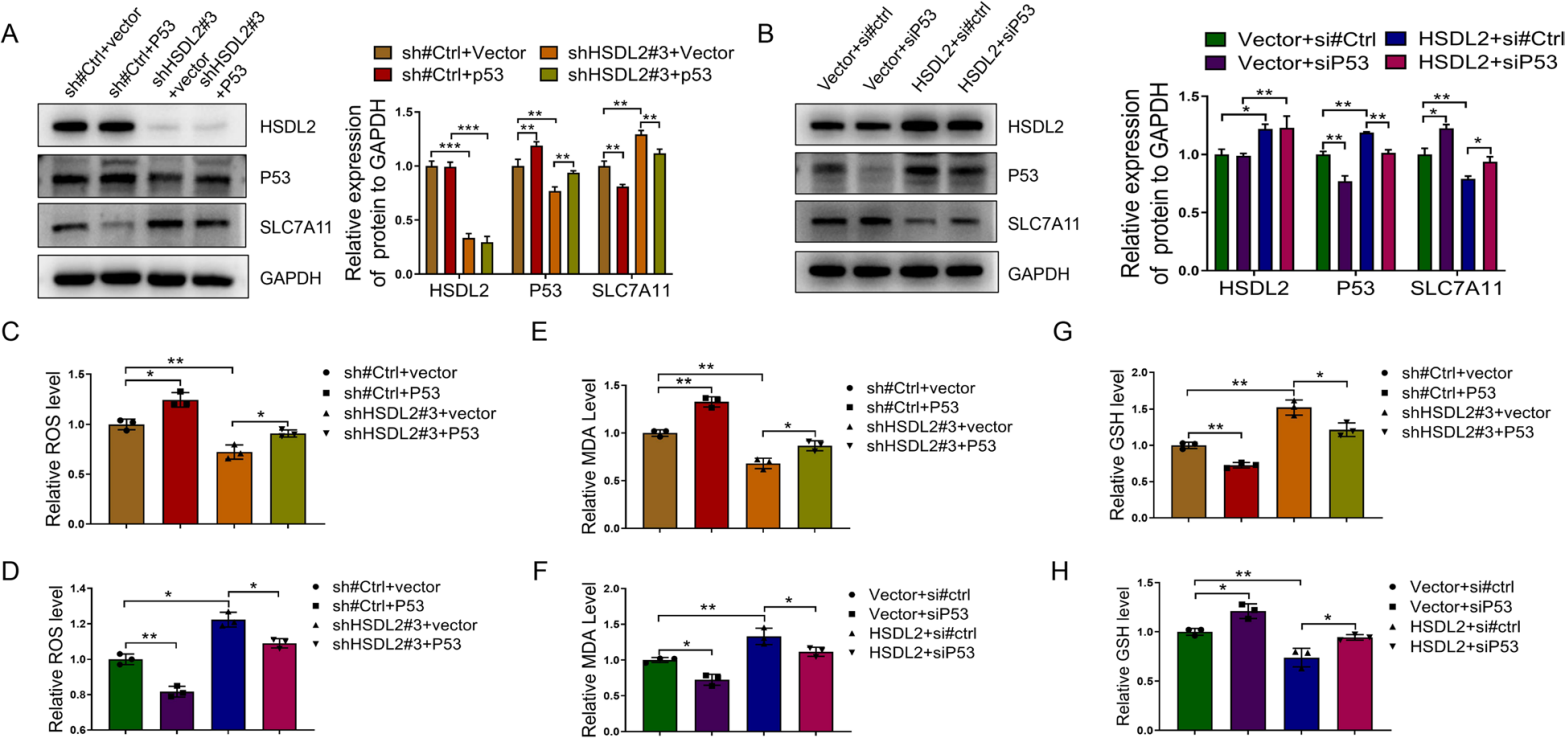
HSDL2 knockdown suppresses ferroptosis by inhibiting the P53-SLC7A11 pathway. **A**, **B** The SLC7A11 expression in the indicated cells were evaluated using western blotting. **C**-**H** The relative reactive oxygen species (ROS) (**C**, **D**), malonaldehyde (MDA) (**E**, **F**), and glutathione (GSH) (**G, H**) levels in the indicated groups were measured using the corresponding kits. Data are presented as mean ± SD (*n=*3). *p < 0.05,**p < 0.01, and ***p < 0.001

HSDL2 knockdown cells were transiently transfected with a p53 overexpression plasmid. P53 overexpression reversed the HSDL2 knockdown-induced upregulation of SLC7A11 (Fig. 5A), increased the levels of lipid ROS (Fig. 5C) and MDA (Fig. 5E), and decreased GSH levels (Fig. 5G) when compared with those in the HSDL2 knockdown group. Conversely, in cells stably overexpressing HSDL2 and transiently transfected with a p53 knockdown plasmid, SLC7A11 and GSH levels increased (Fig. 5B, H), and lipid ROS and MDA levels decreased (Fig. 5D, F). These findings suggest that HSDL2 knockdown suppresses ferroptosis in CCA cells by regulating the p53-SLC7A11 pathway.

### P53 inhibits the transcription of SLC7A11 by binding to its promoter

**Fig. 6:**
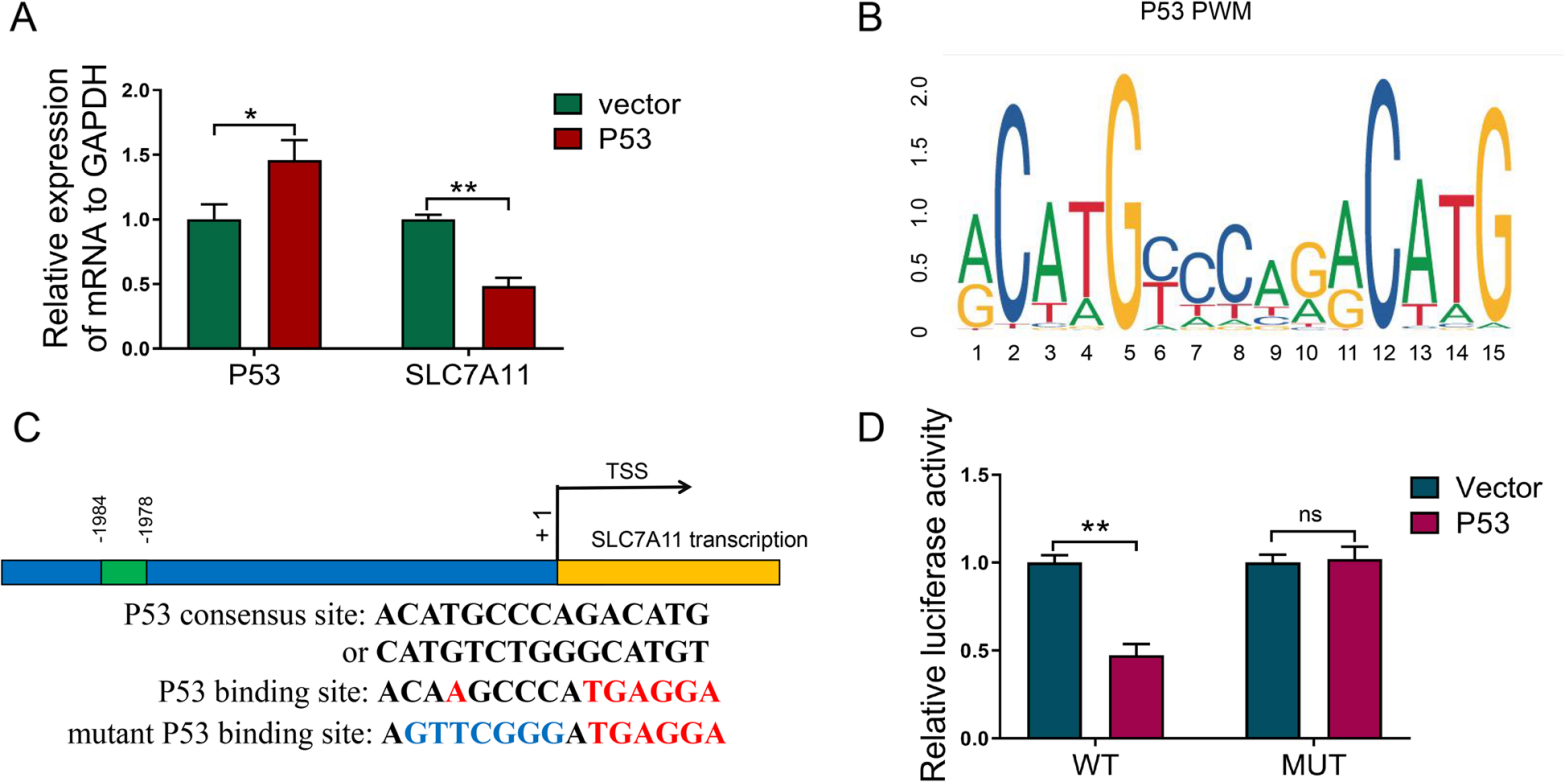
P53 inhibits SLC7A11 transcription by binding to its promoter. **A** After transfecting RBE cells with vector and P53 constructs, the SLC7A11 mRNA level was detected by qRT-PCR. **B** Map of P53 binding site sequence. **C** Schematic illustration of potential P53 binding sites in the SLC7A11 promoter. **D** RBE cells were co-transfected with luciferase reporter constructs containing the wild-type or mutant SLC7A11 promoter and P53 overexpression plasmid or control vector. Data are presented as mean ± SD (*n=*3). **p < 0.01 and ***p < 0.001

P53 overexpression in RBE cells inhibited the mRNA expression of SLC7A11 (Fig. 6A). The p53 binding site in SLC7A11 was predicted using the JASPAR database (https://jaspar.genereg.net/) and ALGGEN PROMO (http://alggen.lsi.upc.es/ cgi-bin/ promo_ v3/ promo) to confirm whether SLC7A11 is a target gene of p53 (Fig. 6B). A set of luciferase reporter vectors carrying full-length or mutated SLC7A11 promoters were established to confirm the binding of SLC7A11 to p53 (Fig. 6C). The luciferase activity of the full-length promoter was significantly lower in p53-overexpressing cells than in positive control cells (Fig. 6D), indicating that p53 directly inhibits SLC7A11 transcription. Mutated SLC7A11 abolished the luciferase activity induced by p53 (Fig. 6D). These results show that p53 transcriptionally represses SLC7A11 by binding to its promoter.

#### HSDL2 knockdown promotes the proliferation, migration, and invasion of CCA cells by inhibiting ferroptosis

**Fig. 7:**
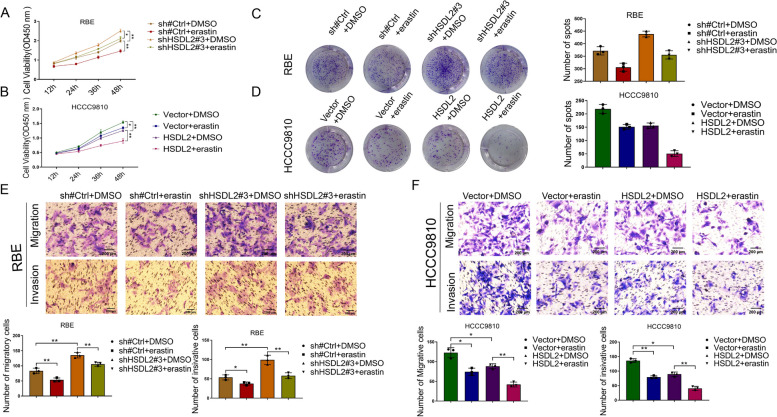
HSDL2 knockdown promotes the proliferation, migration, and invasion of cholangiocarcinoma (CCA) cells by inhibiting ferroptosis. **A**–**D** Cell Counting Kit-8 assay (**A**, **B**) and colony formation assay (**C**, **D**) were applied to assess the effect of erastin (6 µM), which induces ferroptosis, on cell proliferation. (**E**, **F**) A transwell assay was applied to assess the effect of erastin (6 µM) on cell migration and invasion. Data are presented as mean ± SD (*n=*3). **p < 0.01 and ***p < 0.001

The role of ferroptosis in CCA was assessed using erastin, a ferroptosis inducer that inhibits the cystine/glutamate antiporter xCT. Erastin (6 µM) reversed the stimulating effects of HSDL2 knockdown on the proliferation (Fig. 7A, C), migration and invasion (Fig. 7E) of CCA cells. Moreover, erastin potentiated the inhibitory effect of HSDL2 overexpression on the proliferation (Fig. 7B, D), migration and invasion (Fig. 7F) of CCA cells. These results suggest that HSDL2 downregulation promotes CCA progression by inhibiting ferroptosis.

## Discussion

The incidence of CCA, a highly aggressive liver tumor associated with high mortality rates, is increasing [3]. To identify factors that stimulate the proliferation of CCA cells, we focused on HSDL2. HSDL2, from the SDR family, catalyzes the oxidation and reduction of fatty acids, retinoids, steroids, sugars, and vitamins [9]. The critical roles of HSDL2 in the pathogenesis of various diseases, such as cancer, Alzheimer’s disease, and obesity, involve the modulation of signaling and metabolic pathways [30–32]. Ferroptosis, characterized by increased lipid peroxidation, is an iron-dependent type of cell death [17, 18]. Ferroptosis is implicated in the pathogenesis of various diseases and is a potential therapeutic target for cancer [33, 34]. For instance, JUND/linc00976 promotes CCA progression by inhibiting ferroptosis [35]. SHARPIN promotes CCA cell proliferation and inhibits ferroptosis through the p53/SLC7A11/GPX4 pathway [35]. However, the correlation between HSDL2 expression and ferroptosis and the roles of HSDL2 in the pathogenesis of CCA are unclear. Our results showed that HSDL2 was downregulated in CCA and that HSDL2 downregulation promoted the proliferation, migration, and invasion of CCA cells by suppressing ferroptosis.

HSDL2 expression was downregulated in CCA tissues and cell lines, consistent with our previous study using public databases [10]. HSDL2 downregulation is correlated with a poor prognosis. Functional studies demonstrated that HSDL2 downregulation promoted the proliferation, migration, and invasion of CCA cells. Additionally, HSDL2 knockdown stimulated the epithelial-mesenchymal transition (EMT) of CCA cells, evidenced by the decreased expression of the epithelial marker E-cadherin and the increased expression of the interstitial markers N-cadherin, VIM, MMP2, and MMP9 (Supplementary Figure 3A and B). These results show that HSDL2 has a crucial role in CCA progression.

The transcriptome of HSDL2 knockdown CCA cells was sequenced to elucidate the molecular mechanism underlying the HSDL2-mediated regulation of CCA progression. KEGG enrichment analysis showed that the DEGs were significantly enriched in pathways related to cell growth and death and lipid metabolism, in line with previous findings [15, 16]. Further, DEGs enriched in the cell growth and death were markedly enriched in the p53 signaling pathway. The tumor suppressor gene p53 is implicated in the pathogenesis of CCA [36–38]. RNA interference experiments showed that HSDL2 regulates the proliferation, migration, invasion, and EMT (Supplementary Figure 4A and B) of bile duct cancer cells in vivo and in vitro via p53. P53 plays anti-tumor roles by causing apoptosis, aging, and growth arrest. In addition, p53 inhibits cystine uptake and sensitizes cells to ferroptosis by repressing the expression of SLC7A11, a key component of the cystine/glutamate antiporter [20, 39]. xCT controls redox homeostasis and ferroptosis in cancer [40] by inducing the synthesis of GSH and the activation of GPX4. The molecular mechanism by which HSDL2 knockdown inhibits p53 expression is unclear; this was not addressed in this study, which is one limitation, but this is a topic worthy of further study.

Transcriptome analysis revealed that HSDL2 knockdown marginally increased the expression of SLC7A11 (log2 (SH/NC) = 0.65) (*p* = 0.11). Moreover, in the comparisons of the three targeted shRNA/NC shRNA pairs, SLC7A11 was upregulated in the knockdown group for two pairs (FPKM: SH1/NC1 = 1.98:0.88, SH3/NC3 = 2.59:1.31), while SLC7A11 was downregulated in the knockdown group for the other pair (FPKM: SH2/NC2 = 0.77:0.94). This discrepancy may be due to differences between samples.

SLC7A11, the catalytic subunit of the xCT system, is upregulated in several cancers and is an indicator of antiporter xCT activity [41]. We searched for SLC7A11 data in online databases. GEPIA2 data indicated that SLC7A11 was upregulated in CCA cells, in line with IF data for CCA tissues. In addition, analysis of the GEPIA datasets suggested that HSDL2 expression was negatively correlated with SLC7A11 expression. Subsequent cell experiments also revealed that HSDL2 can negatively regulate SLC7A11. The absence of the cysteine-glutathione-GpX4 axis and the accumulation of lipid peroxides are characteristics of ferroptosis [29], which leads to a decrease in glutathione levels and an increase in lipid reactive oxygen species and MDA levels. HSDL2 knockdown increased intracellular GSH levels and decreased lipid ROS and MDA levels, and the increase in p53 expression reversed these effects. These changes in ferroptosis biochemical indicators were sufficient to explain the regulation of HSDL2 on ferroptosis levels, although we did not further explore the changes in GPX4 and labile iron pool. Instead, we focused on SLC7A11, a molecule associated with ferroptosis. P53 inhibits the transcription of SLC7A11 by binding to its promoter. Precisely because P53 is the upstream transcriptional suppressor of SLC7A11, it will have a greater impact on SLC7A11 expression than HSDL2, and further affect the relevant indicators of ferroptosis, such as MDA, ROS and GSH. In addition, changes in the expression level of p53 can partially reverse the effects of HSDL2 on SLC7A11 expression (Fig. 5A and B) and ferroptosis level (Fig. 5C - H), which suggests that HSDL2 regulates ferroptosis through the p53-SLC7A11 axis. Ferroptosis was induced in HSDL2 knockdown CCA cells using erastin. Furthermore, ferroptosis reversed the stimulating effect of HSDL2 knockdown on the proliferation, migration, and invasion of CCA cells.

Based on the above results, we conclude that HSDL2 is an effective target for clinical diagnosis and treatment of cholangiocarcinoma, and more experiments are needed to prove its value. However, there are some shortcomings to this study. For example, the mechanism linking HSDL2 and p53 is unclear, in vivo experiments related to ferroptosis need to be optimized, and tumor organoid models need to be developed to confirm its clinical therapeutic value. Molecular immunotherapy plays a crucial role in the clinical treatment of tumors. In the future, molecular-based cancer research strategies can be used to preliminarily screen molecules that may play a role in targeted therapies.

## Conclusion

The HSDL2/p53/SLC7A11 axis underlies CCA progression. Thus, HSDL2 is a potential prognostic marker and a therapeutic target for CCA.

### Supplementary Information

**Additional file 1: Supplementary Table 1.** Antibodies and their information. **Supplementary Table 2.** Reagents and their information . **Supplementary Table 3.** Specific sequences of lentiviral plasmids (5’→3’). **Supplementary Table 4.** Specific sequences of siRNAs/shRNA (5’→3’). **Supplementary Table 5.** Primer sequences(5’→3’). **Supplementary Figure 1.** The infective efficiency of lentivirus during the construction of stable cell lines. **Supplementary Figure 2.** Detection of p53 knockout and overexpression. **Supplementary Figure 3.** HSDL2 can affect the epithelial-to-mesenchymal (EMT) of cholangiocarcinoma (CCA) cells. **Supplementary Figure 4.** p53 can affect the epithelial-to-mesenchymal (EMT) of cholangiocarcinoma (CCA) cells.

**Additional file 2. **

## Data Availability

The data are available from the corresponding author. Transcriptome data related to this experiment are stored in the GEO database (GSE225900).

## Abbreviations

CCA: Cholangiocarcinoma
IHC: Immunohistochemistry
IF: Immunofluorescence
GSH: Glutathione
MDA: Malondialdehyde
OS: Overall survival
DFS: Disease-free survival
EMT: Epithelial-mesenchymal transition
