# Supplementary material for: HSDL2 knockdown promotes the progression of cholangiocarcinoma by inhibiting ferroptosis through the P53/SLC7A11 axis
Source: World J Surg Oncol. 2023 Sep 18;21:293. doi: 10.1186/s12957-023-03176-6 (PMC10506268; doi:10.1186/s12957-023-03176-6)
Supplement: Supplementary file 1 — Additional file 1: Supplementary Table 1. Antibodies and their information. Supplementary Table 2. Reagents and their information . Supplementary Table 3. Specific sequences of lentiviral plasmids (5’→3’). Supplementary Table 4. Specific sequences of siRNAs/shRNA (5’→3’). Supplementary Table 5. Primer sequences(5’→3’). Supplementary Figure 1. The infective efficiency of lentivirus during the construction of stable cell lines. Supplementary Figure 2. Detection of p53 knockout and overexpression. Supplementary Figure 3. HSDL2 can affect the epithelial-to-mesenchymal (EMT) of cholangiocarcinoma (CCA) cells. Supplementary Figure 4. p53 can affect the epithelial-to-mesenchymal (EMT) of cholangiocarcinoma (CCA) cells. [file 12957_2023_3176_MOESM1_ESM.doc]

**Supplementary materials**

**Supplementary Table 1. Antibodies and their information**

| Antibody information |
| --- |
| **western blotting**  HSDL2 (1:1000, Abcam, USA, ab181174)  E-cadherin (1:2000, Proteintech, China, 60335-1-Ig)  N-cadherin (1:2000, Proteintech, China, 66219-1-Ig)  Vimentin (1:1000, Proteintech, China, 60330-1-Ig)  MMP2 (1:1000, Proteintech, China, 66366-1-Ig)  MMP9 (1:1000, Proteintech, China, 10375-2-AP)  P53 (1:1000, Proteintech, China, 60283-2-Ig)  SLC7A11(xCT) (1:1000, Abcam, USA, ab175186)  GAPDH (1:10000, CST, USA, 5174)  goat anti-rabbit (1:10000, biosharp,China,BL003A)  goat anti-mouse ( 1:10000, biosharp,China,BL001A)  **immunofluorescence**  SLC7A11(xCT) (1:200, Abcam, USA, ab37185)  CoraLite594-conjugated Goat Anti-Rabbit IgG(H+L)  (1:200, Proteintech, China, SA00013-4)  **IHC**  HSDL2 (1:200, Abcam, USA, ab181174) |

**Supplementary Table 2. Reagents and their information**

| Reagent information |
| --- |
| Puromycin (Solarbio, China, P8230)  lipofectamine 2000 (Thermo, USA, 11668019 )  TRIzol reagent (Thermo, USA, 15596018 )  the RevertAid First Strand cDNA synthesis kit (Thermo, USA, K1622)  real-time quantitative PCR assay kit (Takara Bio, Japan, RR820A)  Cell counting kit-8(CCK8) (Biosharp, China, BS350B)  BeyoClickTM 5-ethynyl-2′-deoxyuridine  (EdU) cell proliferation kit Alexa Fluor 555 (Beyotime, China, (C0075S) |

| lentiviral plasmids sequences |
| --- |
| shHSDL2#1 GCAGCAAAGGATGGAGCAAAT  shHSDL2#2 GCACCTACCTTGCATCTAAAG  shHSDL2#3 GCACTGTGCTTATACCATTGC |

**Supplementary Table 3**. Specific sequences of lentiviral plasmids (5’→3’)

**Supplementary Table 4**. Specific sequences of siRNAs/shRNA (5’→3’)

| siRNAs/shRNA sequences |
| --- |
| siP53 GCAUGAACCGGAGGCCCAU  shHSDL2#1 GCAGCAAAGGATGGAGCAAAT  shHSDL2#3 GCACTGTGCTTATACCATTGC |

**Supplementary Table5**. Primer sequences(5’→3’)

| Gene primer sequences |
| --- |
| HSDL2 F: 5’-ATGTTACCCAACACCGGGAG-3’  R:5’-GCTTTCAATGCAATAGCTTTGCC-3’  P53 F:5’-CAGCACATGACGGAGGTTGT-3’  R:5’-TCATCCAAATACTCCACACGC-3’  SLC7A11 F:5’-TCTCCAAAGGAGGTTACCTGC-3’  R:5’-AGACTCCCCTCAGTAAAGTGAC-3’  GAPDH F:5’-GAGAAGTATGACAACAGCCTCAA-3’  R:5’-GCCATCACGCCACAGTTT-3’ |

**Supplementary Figure 1. The infective efficiency of lentivirus during the construction of stable cell lines.**

**
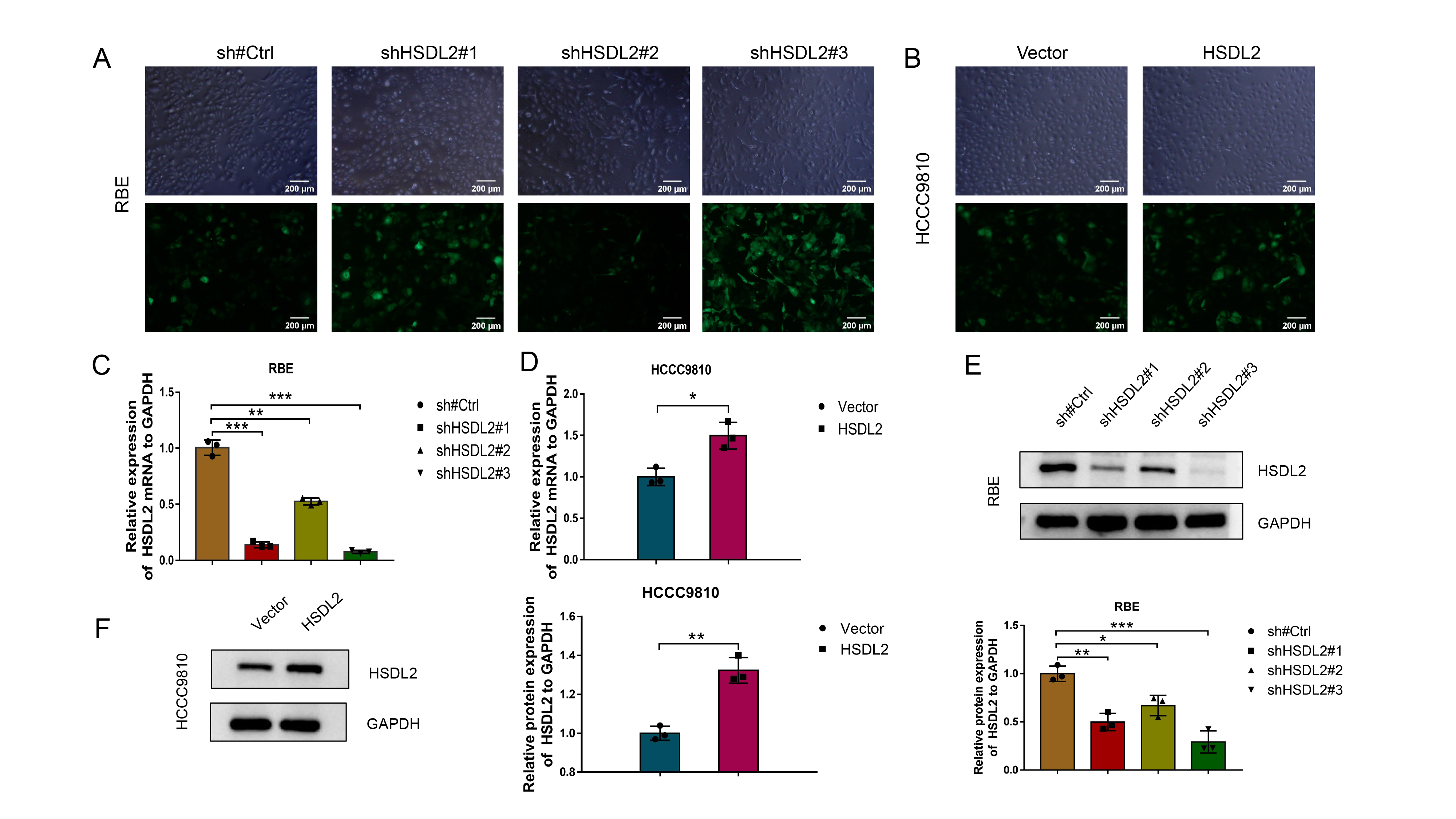
**

(A, B) The efficiency of lentivirus infection in RBE (A) and HCCC9810 (B) cholangiocarcinoma (CCA) cells. (C–F) Transfection efficiencies were assessed by qRT-PCR (C, D) and western blotting (E, F). Data are presented as mean ± SD (n=3). *p < 0.05, **p < 0.01, and ***p < 0.001.

**Supplementary Figure 2. Detection of p53 knockout and overexpression**

**
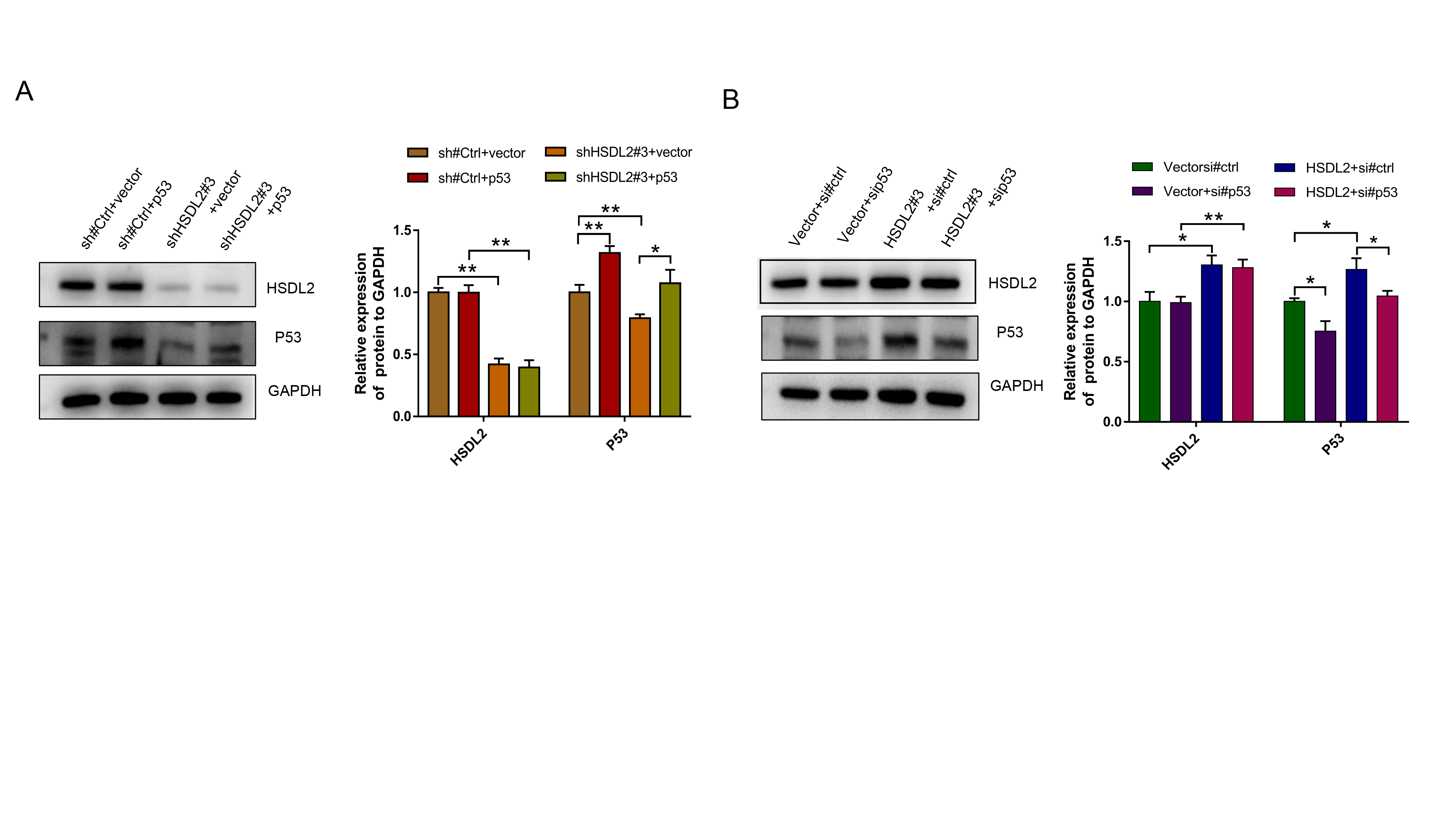
**

(A, B) Western blotting analyses were applied to examine the effects of p53 overexpression(A) or knockdown (B). Data are presented as mean ± standard deviation (n=3). *p < 0.05, **p < 0.01, and ***p < 0.001.

**Supplementary Figure 3. HSDL2 can affect the epithelial-to-mesenchymal (EMT) of cholangiocarcinoma (CCA) cells.**

**
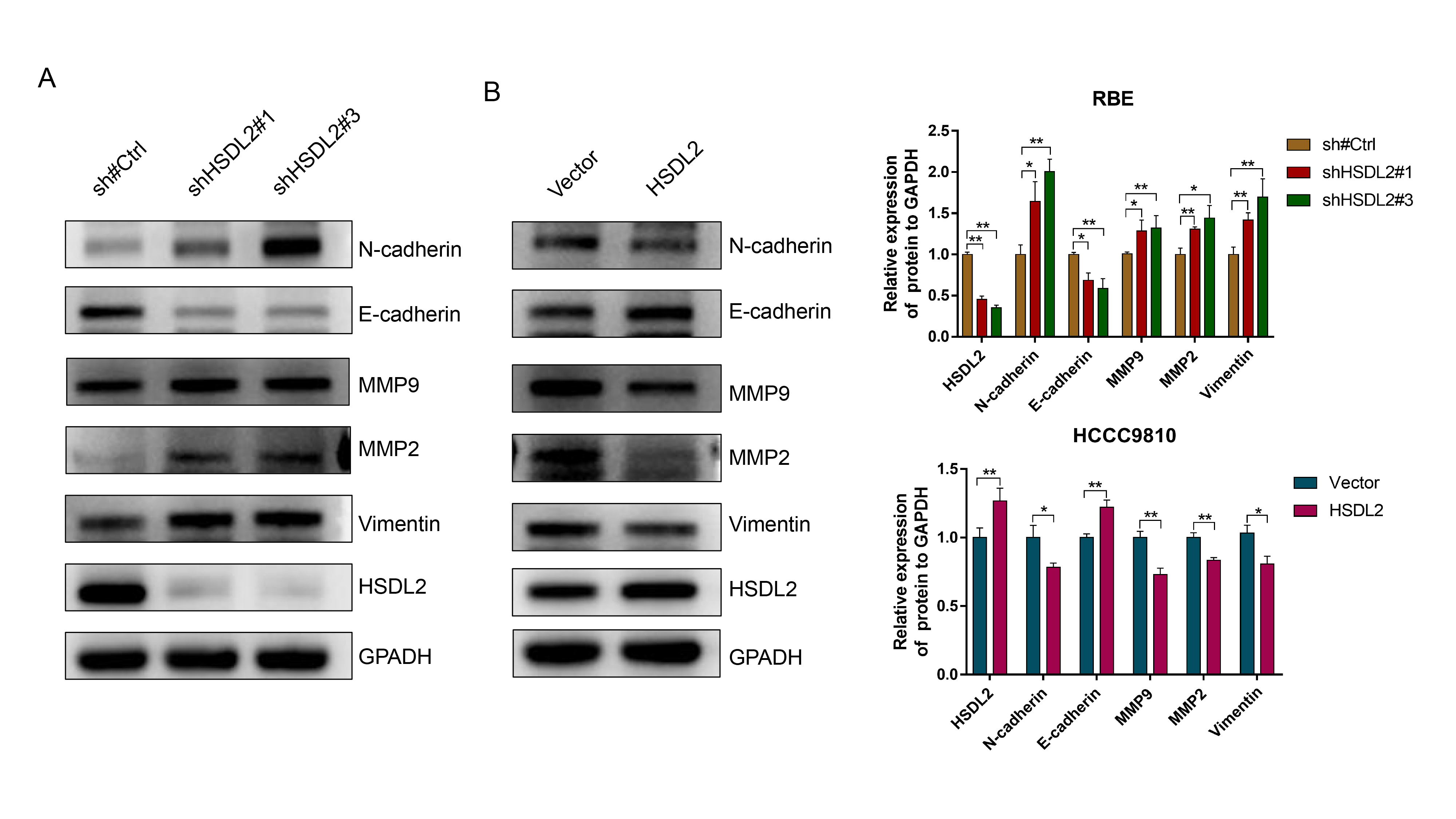
**

(A, B) Western blotting was applied to evaluate the effects of HSDL2 knockdown(A) or overexpression (B) on the expression levels of E-cadherin, N-cadherin, VIM, MMP2, and MMP9. Data are presented as mean ± SD (n=3). *p < 0.05, **p < 0.01, and ***p < 0.001.

**Supplementary Figure 4. p53 can affect the epithelial-to-mesenchymal (EMT) of cholangiocarcinoma (CCA) cells.**

**
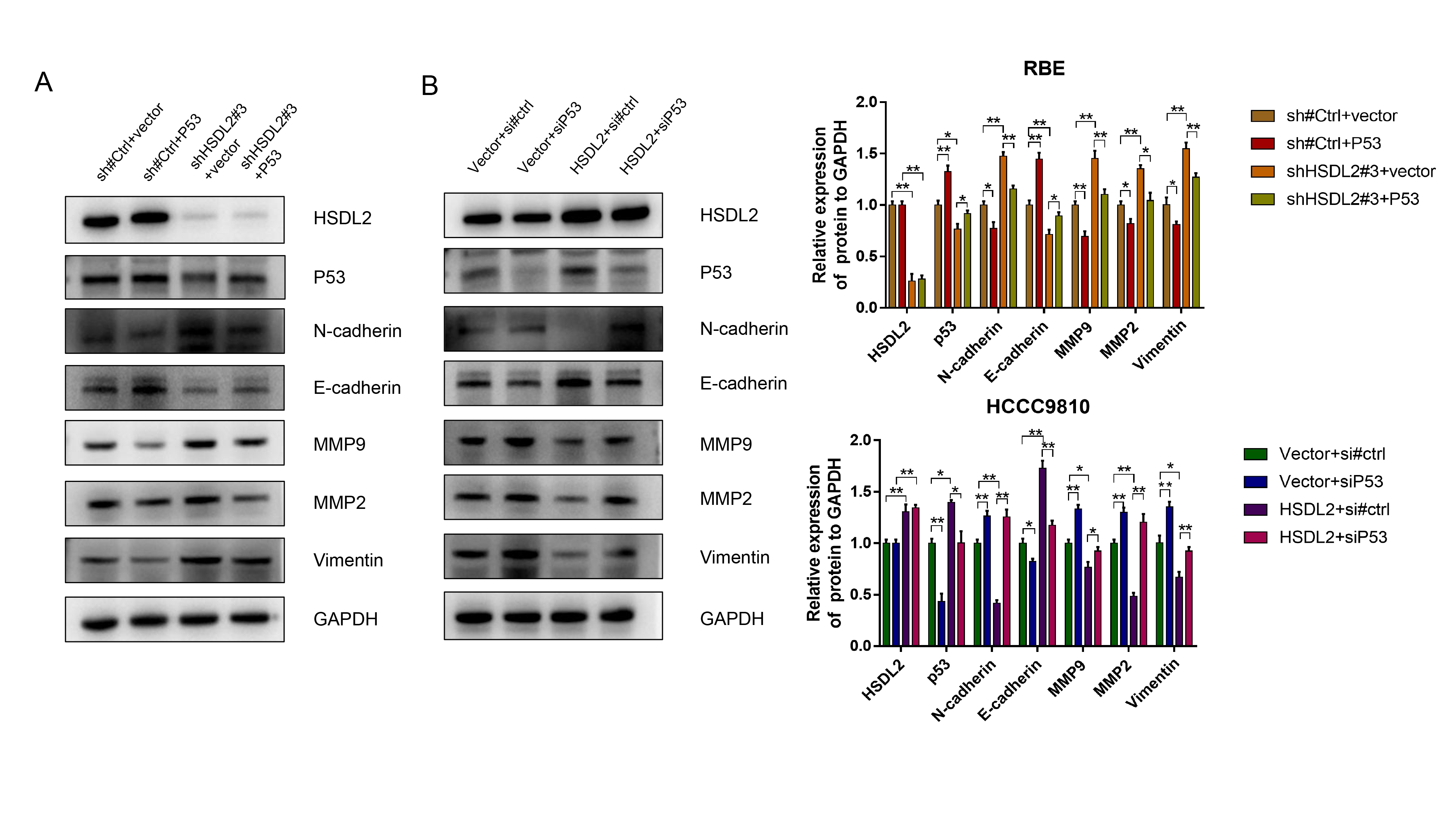
**

(A, B) Western blotting was applied to determine the impact of P53 interference on the expression levels of E-cadherin, N-cadherin, VIM, MMP2, and MMP9 after p53 overexpression(A) and knockdown (B). Data are presented as mean ± SD (n=3). *p < 0.05, **p < 0.01, and ***p < 0.001.
