# Supplementary material for: HSDL2 knockdown promotes the progression of cholangiocarcinoma by inhibiting ferroptosis through the P53/SLC7A11 axis
Source: World J Surg Oncol. 2023 Sep 18;21:293. doi: 10.1186/s12957-023-03176-6 (PMC10506268; doi:10.1186/s12957-023-03176-6)
Supplement: Supplementary file 2 — Additional file 2. [file 12957_2023_3176_MOESM2_ESM.pptx]

## Slide 1
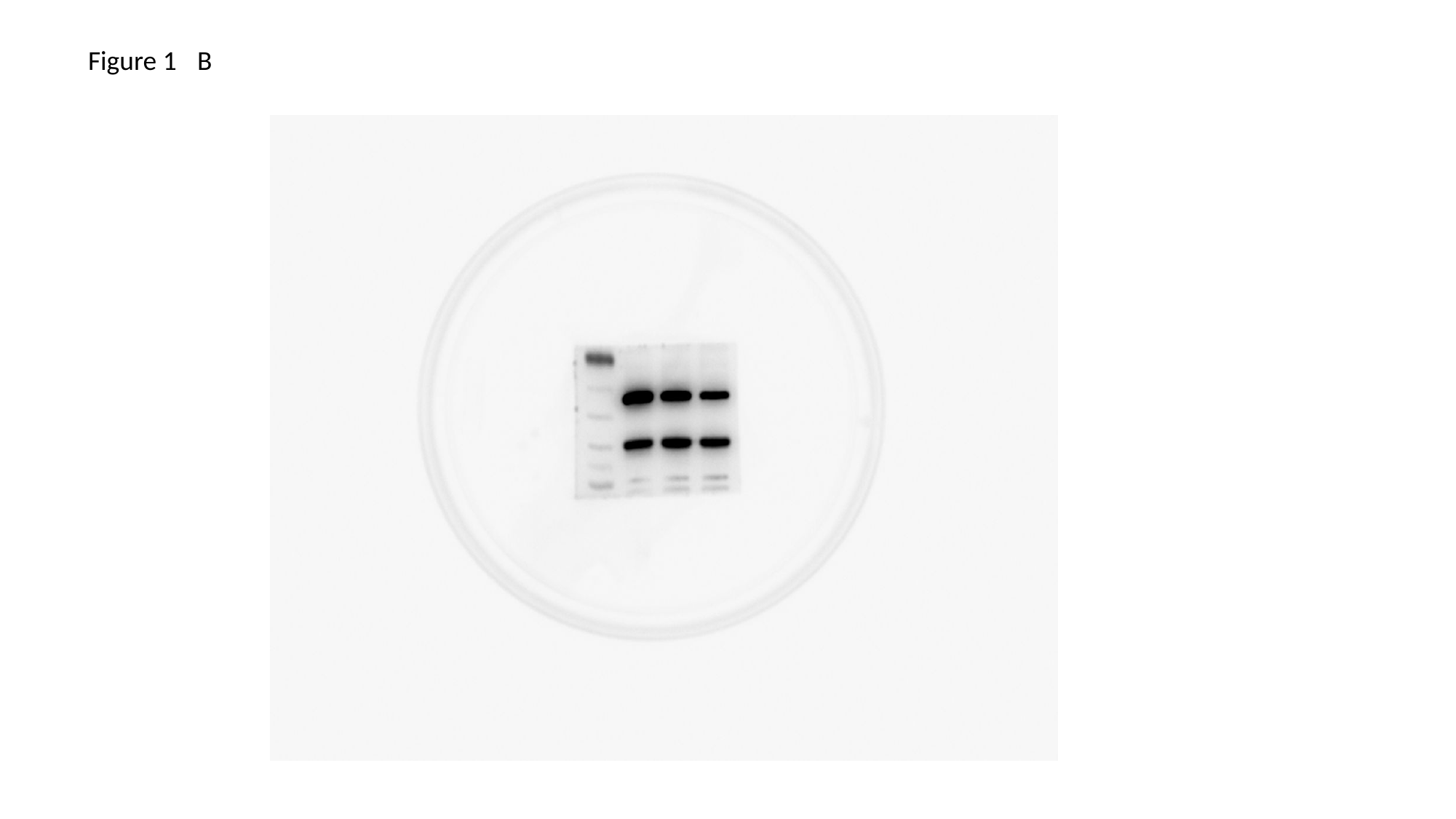

Figure 1	B

## Slide 2
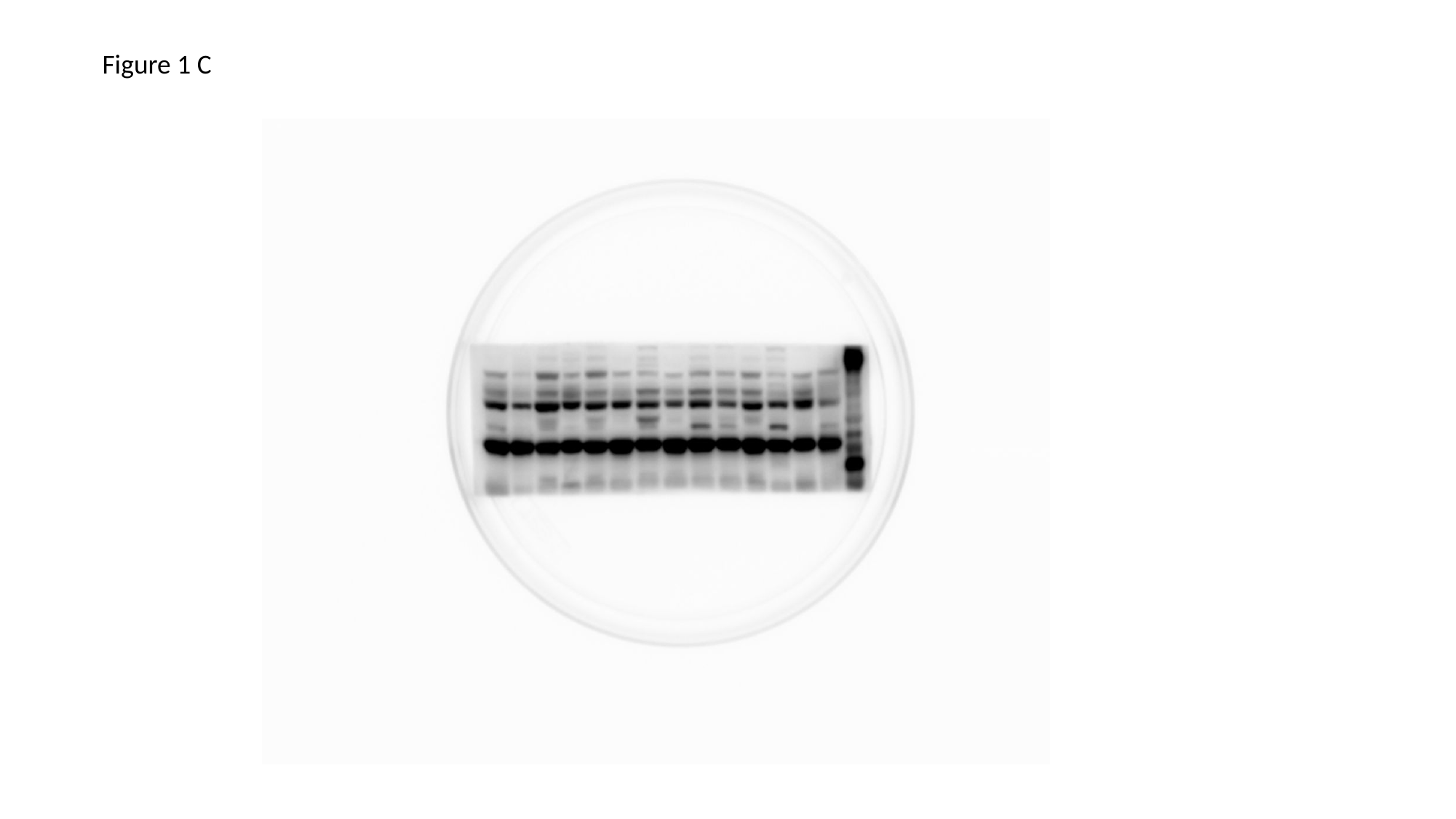

Figure 1 C

## Slide 3
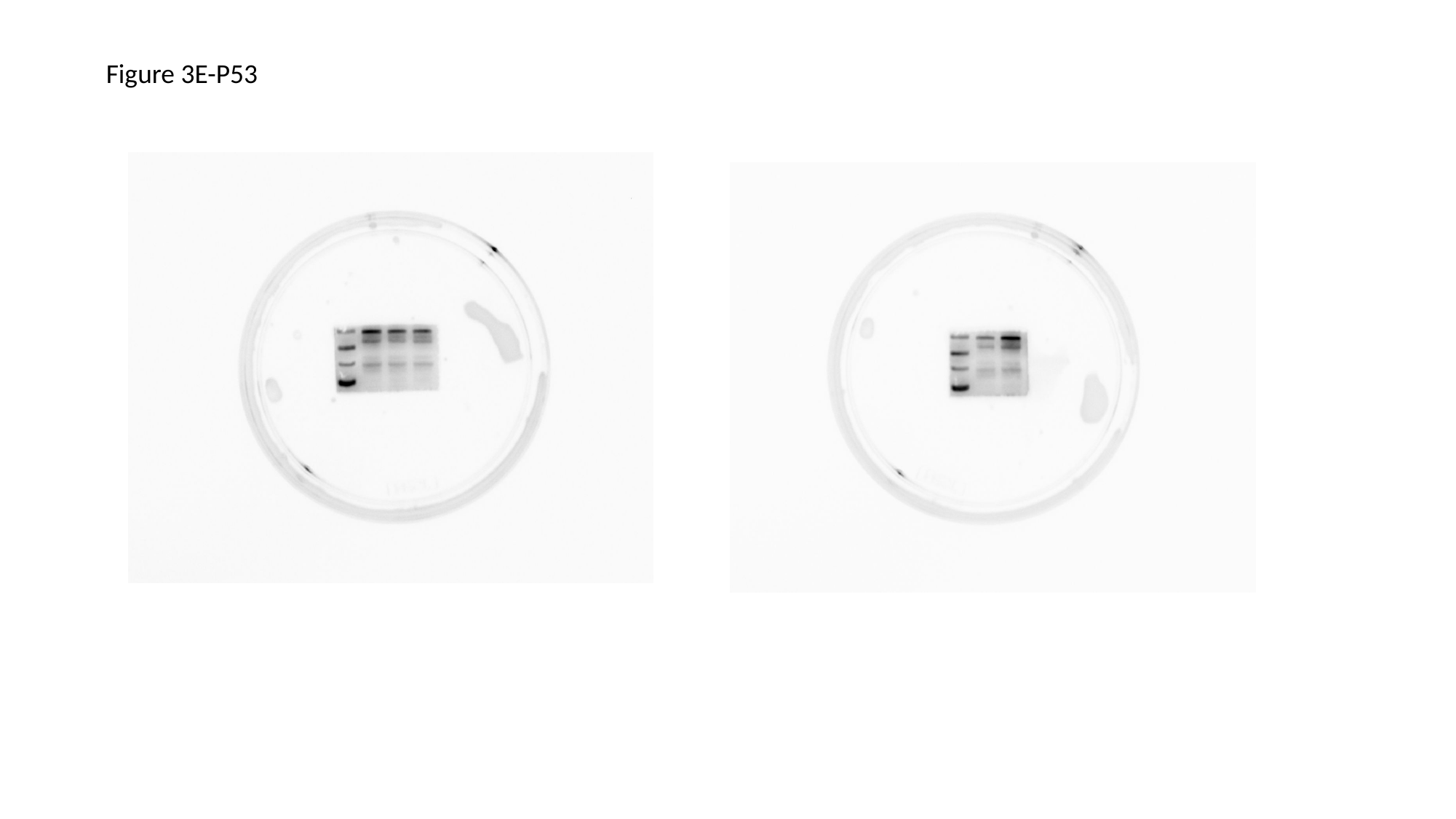

Figure 3E-P53

## Slide 4
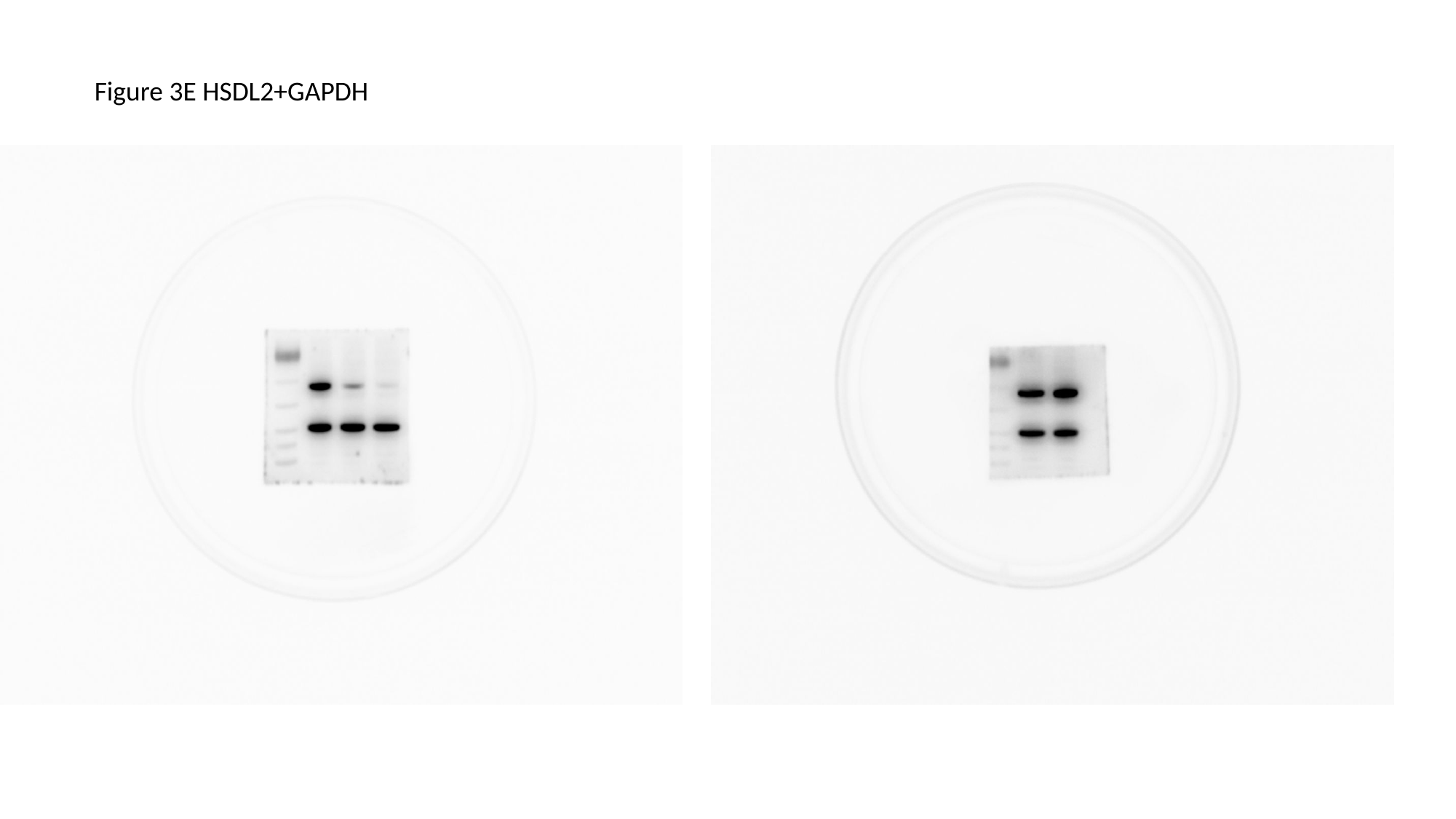

Figure 3E HSDL2+GAPDH

## Slide 5
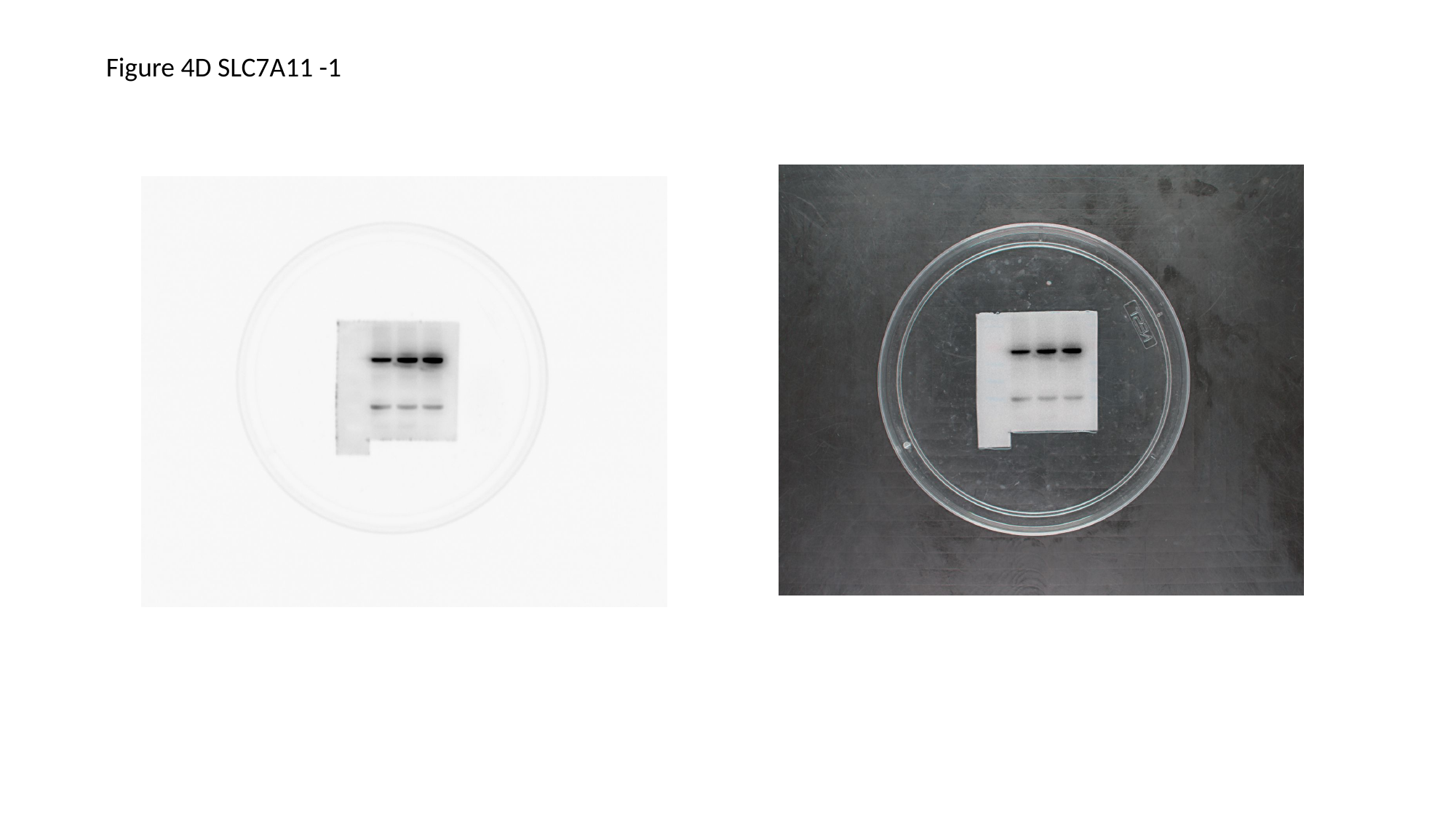

Figure 4D SLC7A11 -1

## Slide 6
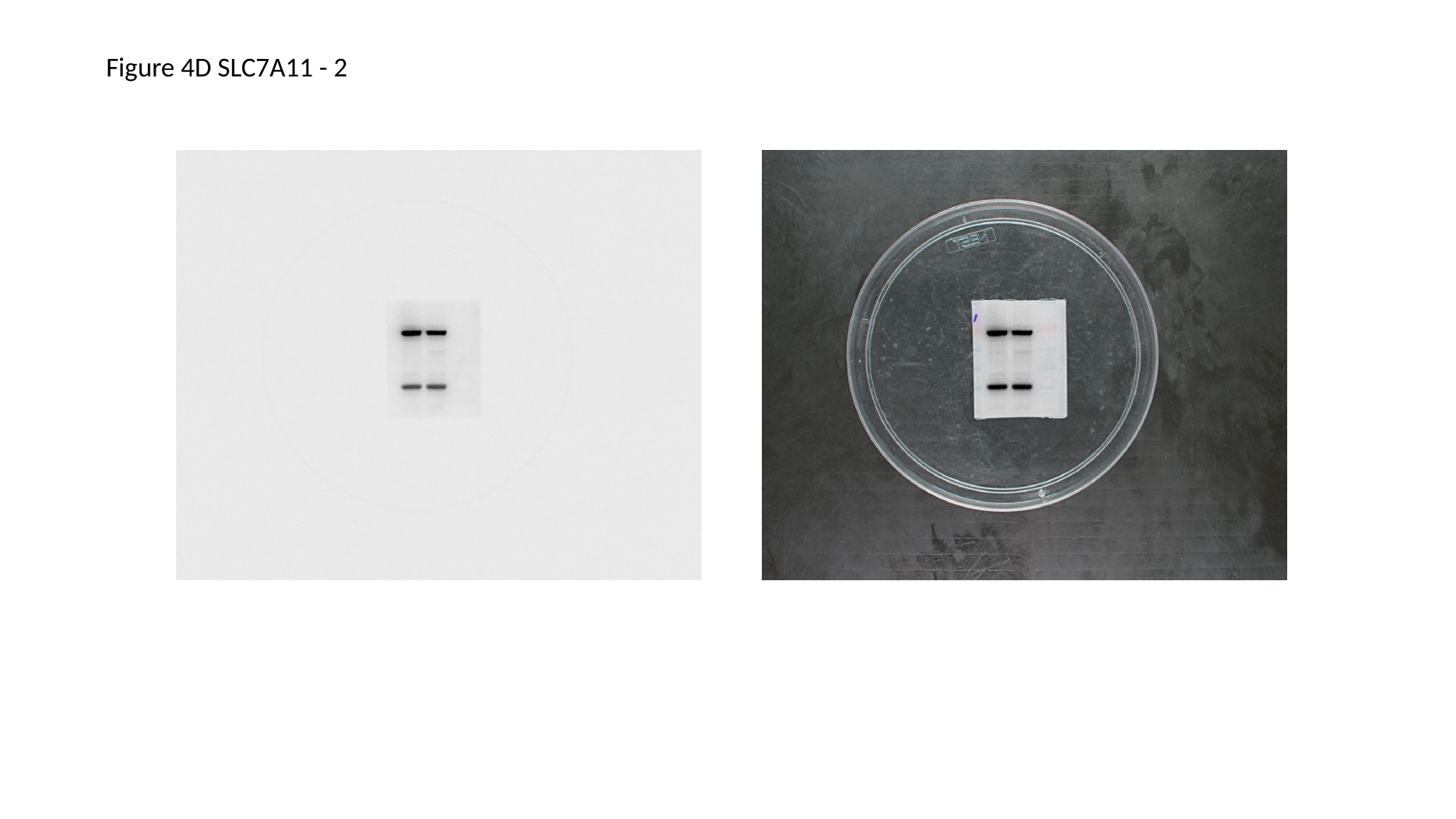

Figure 4D SLC7A11 - 2

## Slide 7
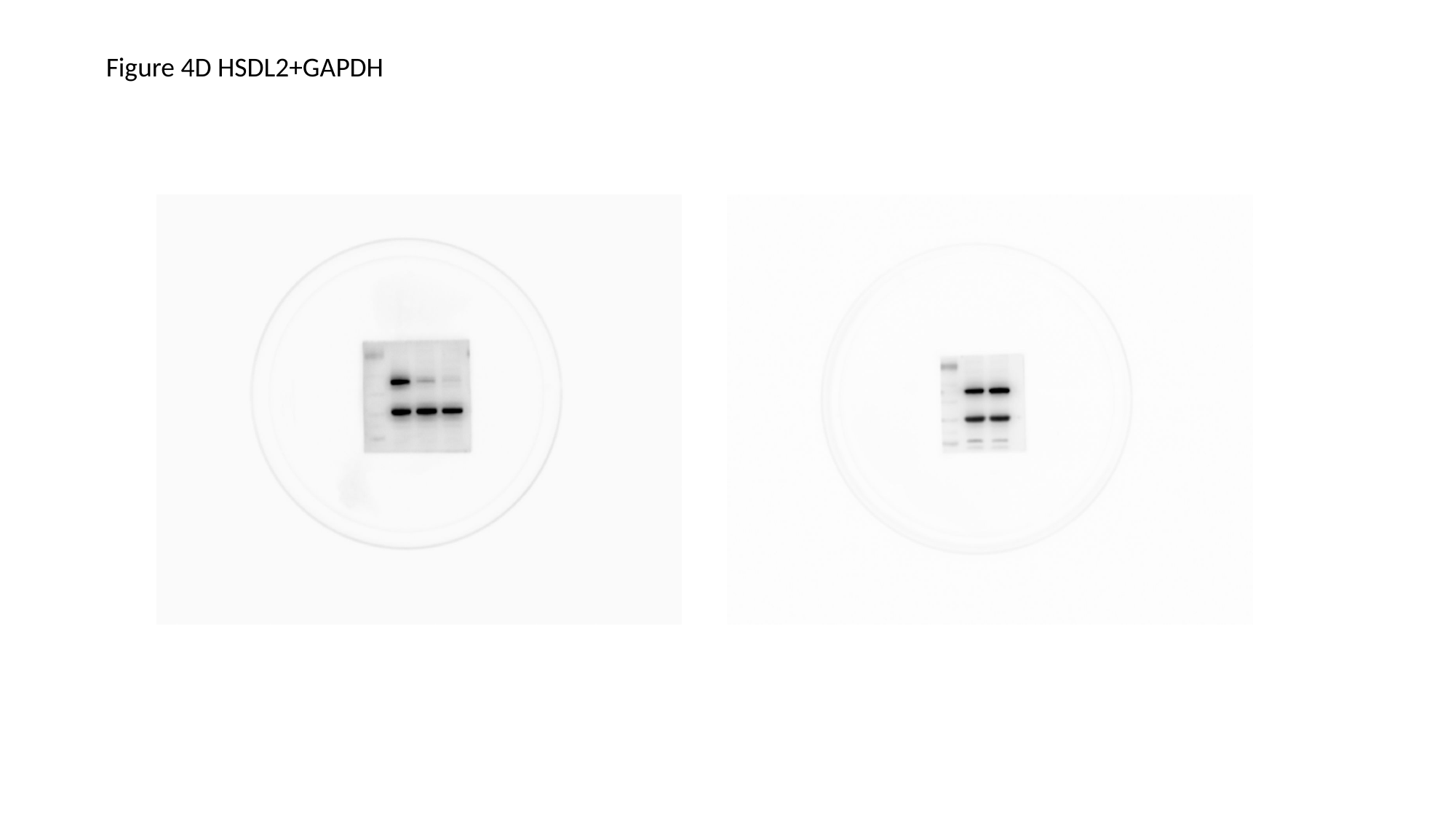

Figure 4D HSDL2+GAPDH

## Slide 8
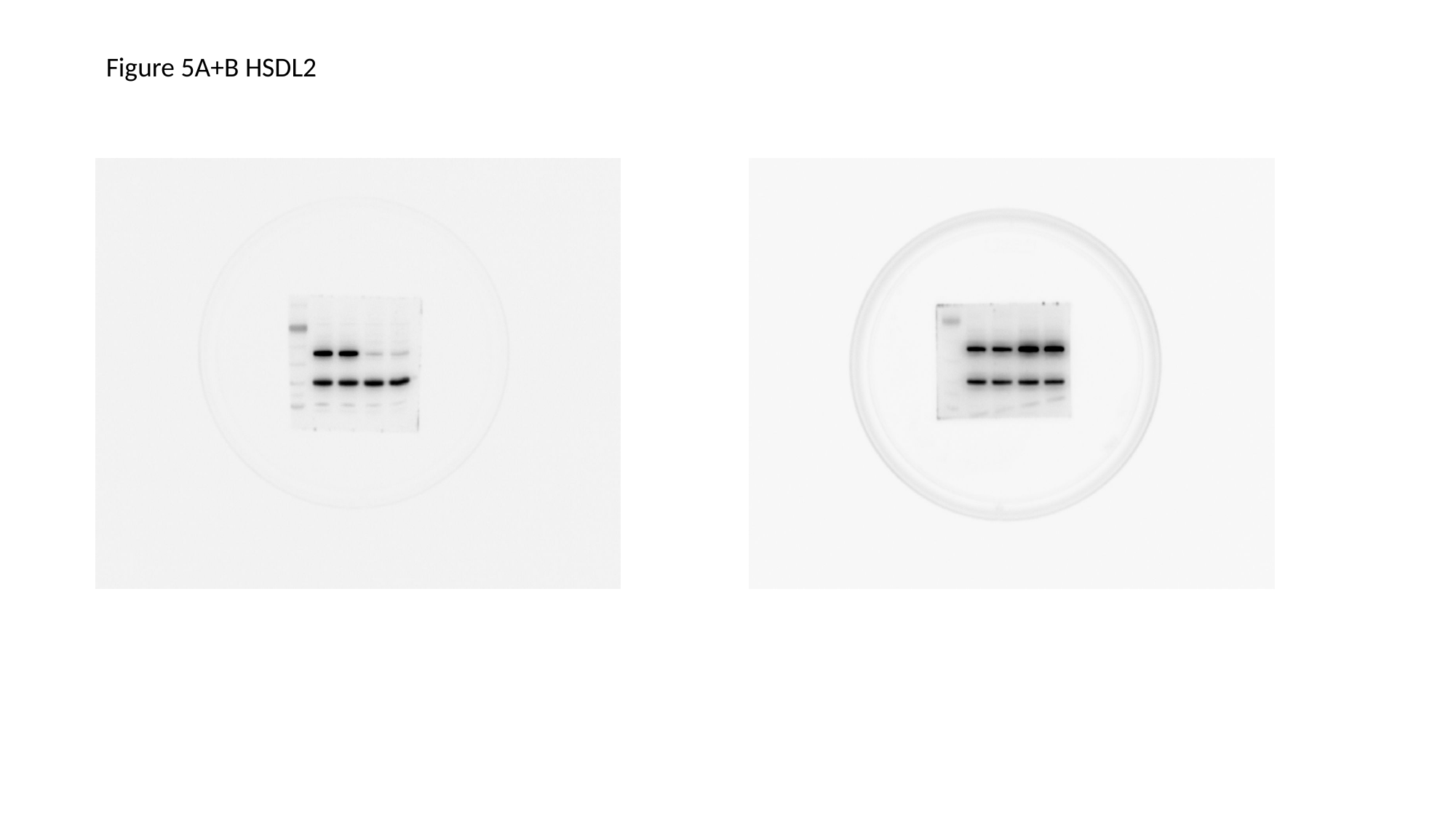

Figure 5A+B HSDL2

## Slide 9
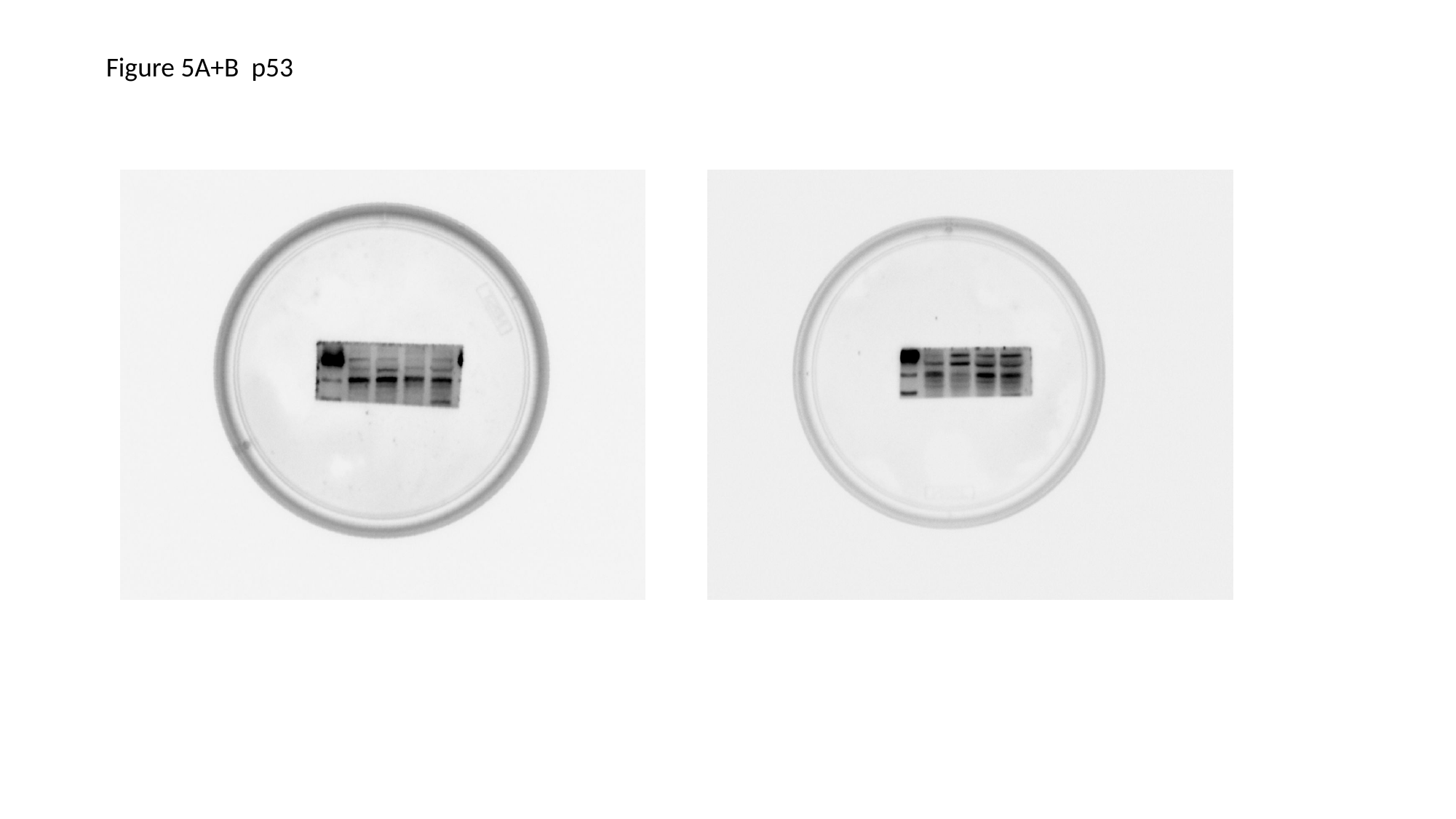

Figure 5A+B p53

## Slide 10
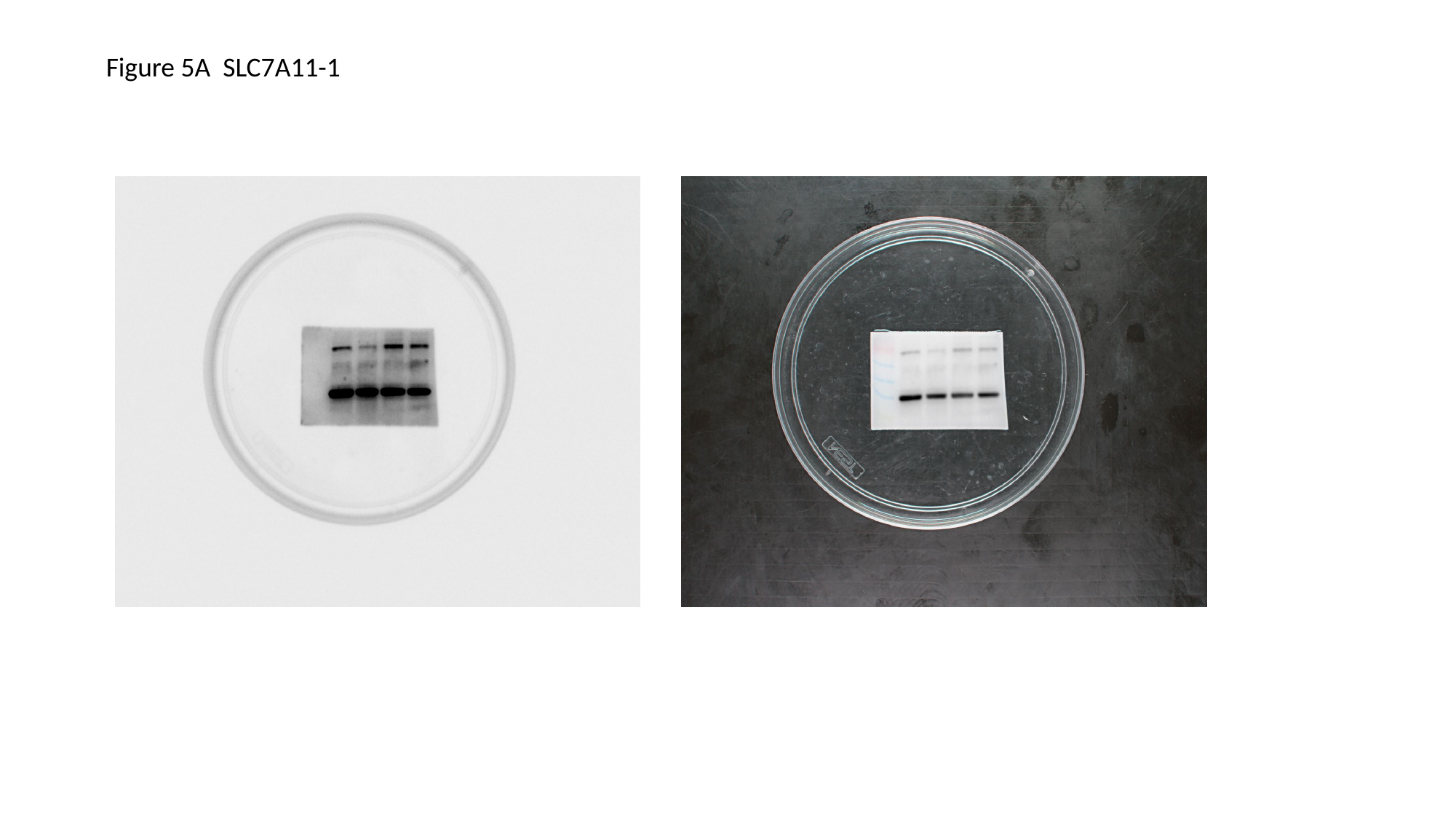

Figure 5A SLC7A11-1

## Slide 11
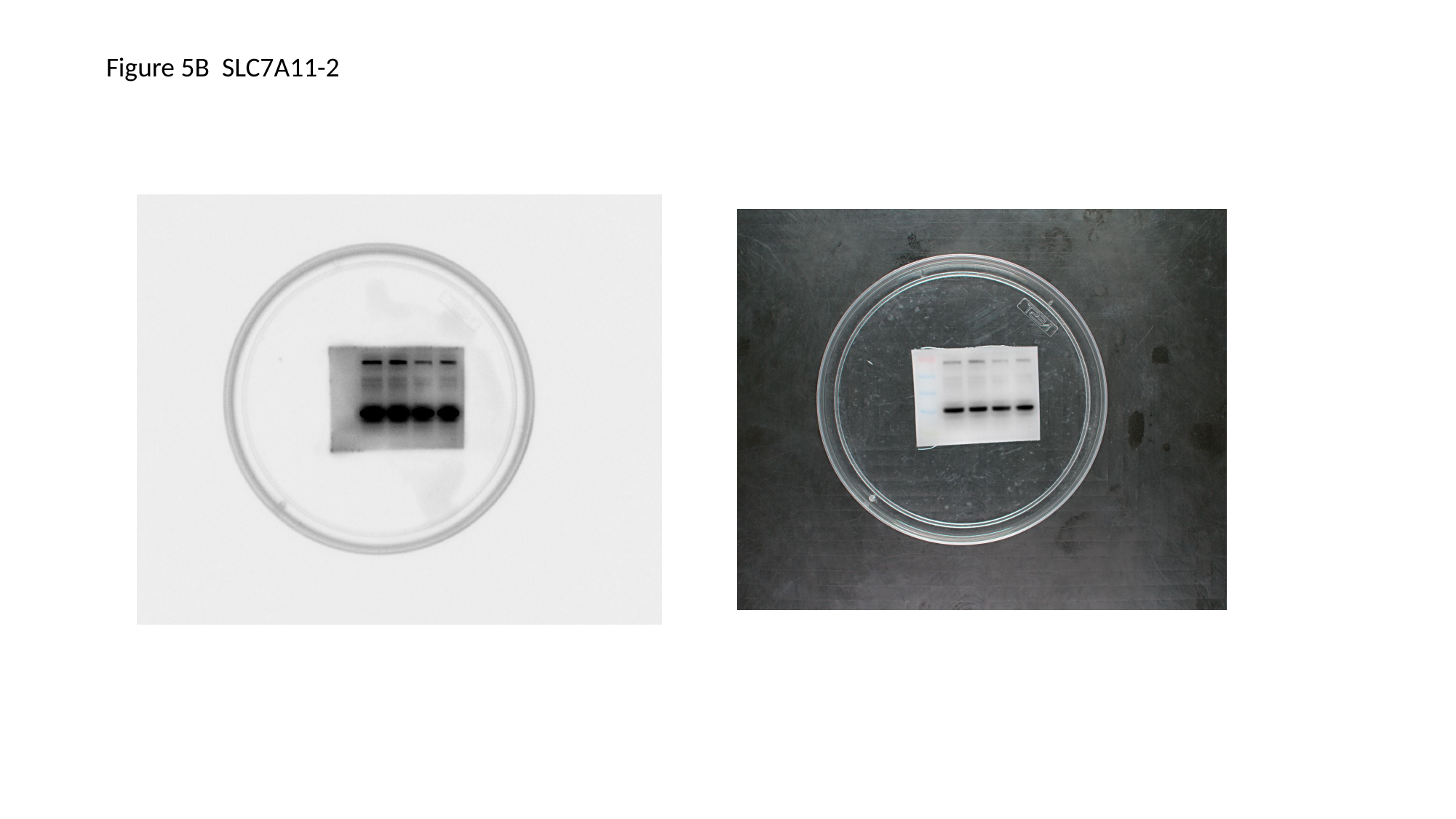

Figure 5B SLC7A11-2

## Slide 12
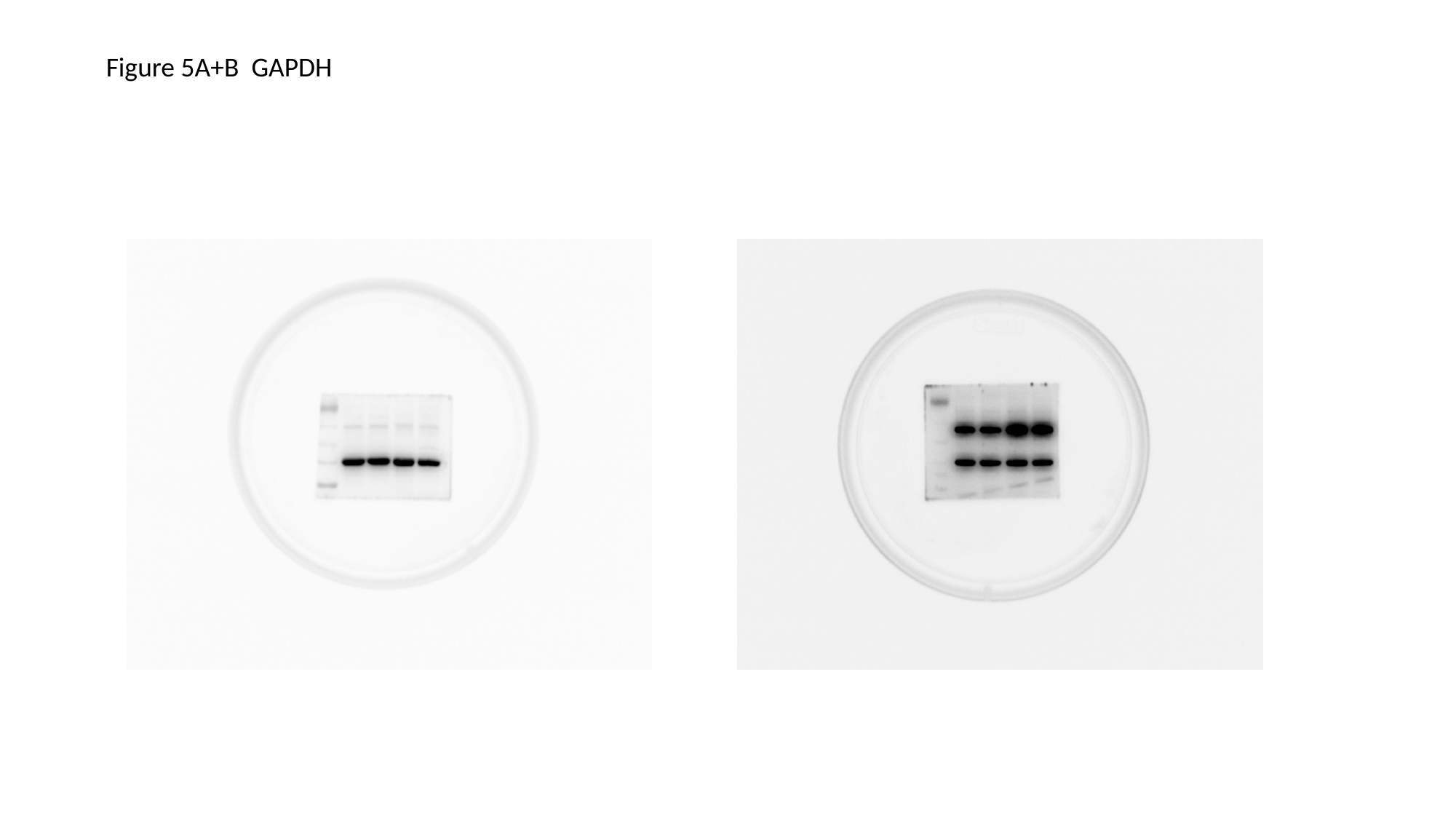

Figure 5A+B GAPDH

## Slide 13
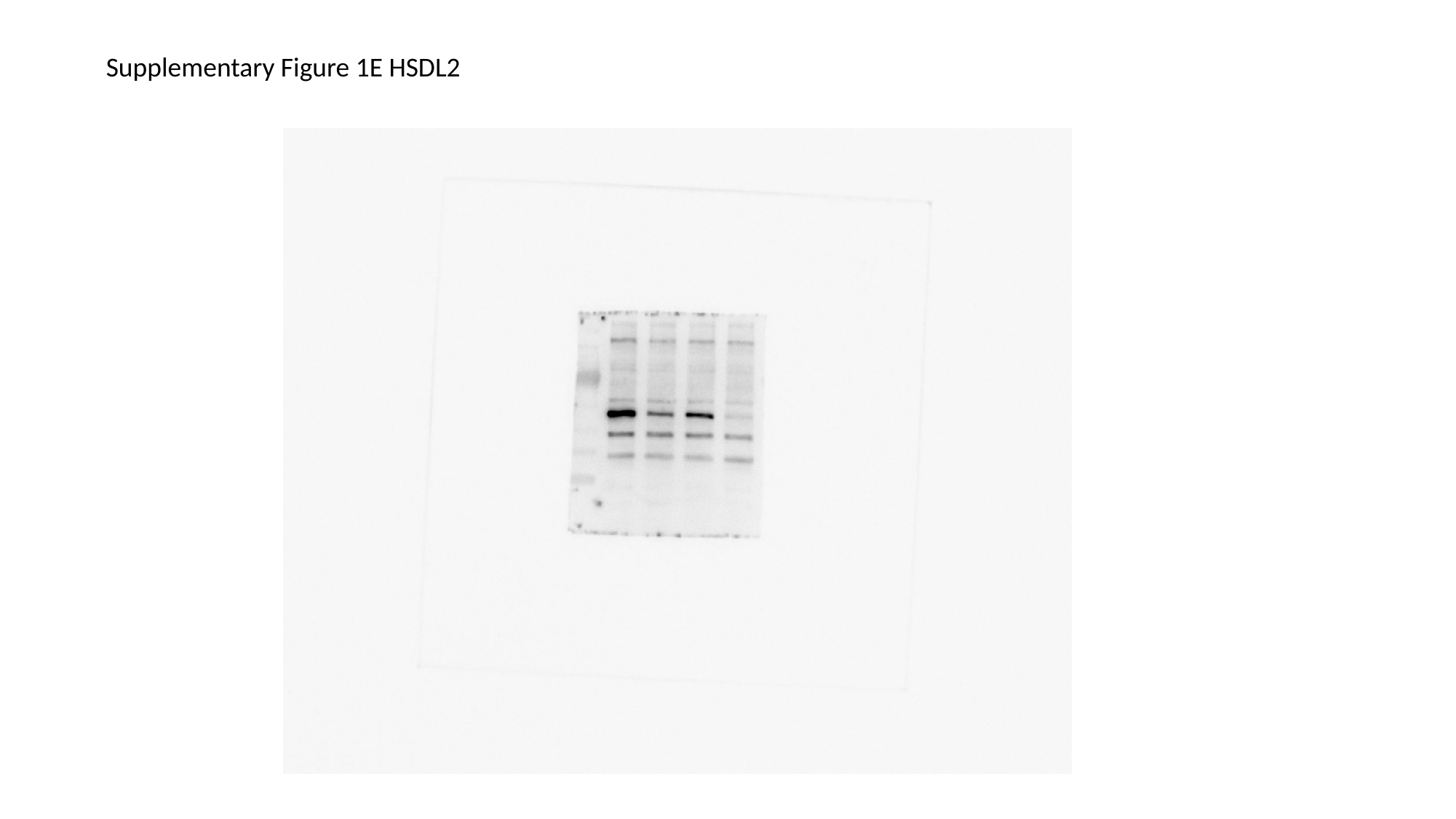

Supplementary Figure 1E HSDL2

## Slide 14
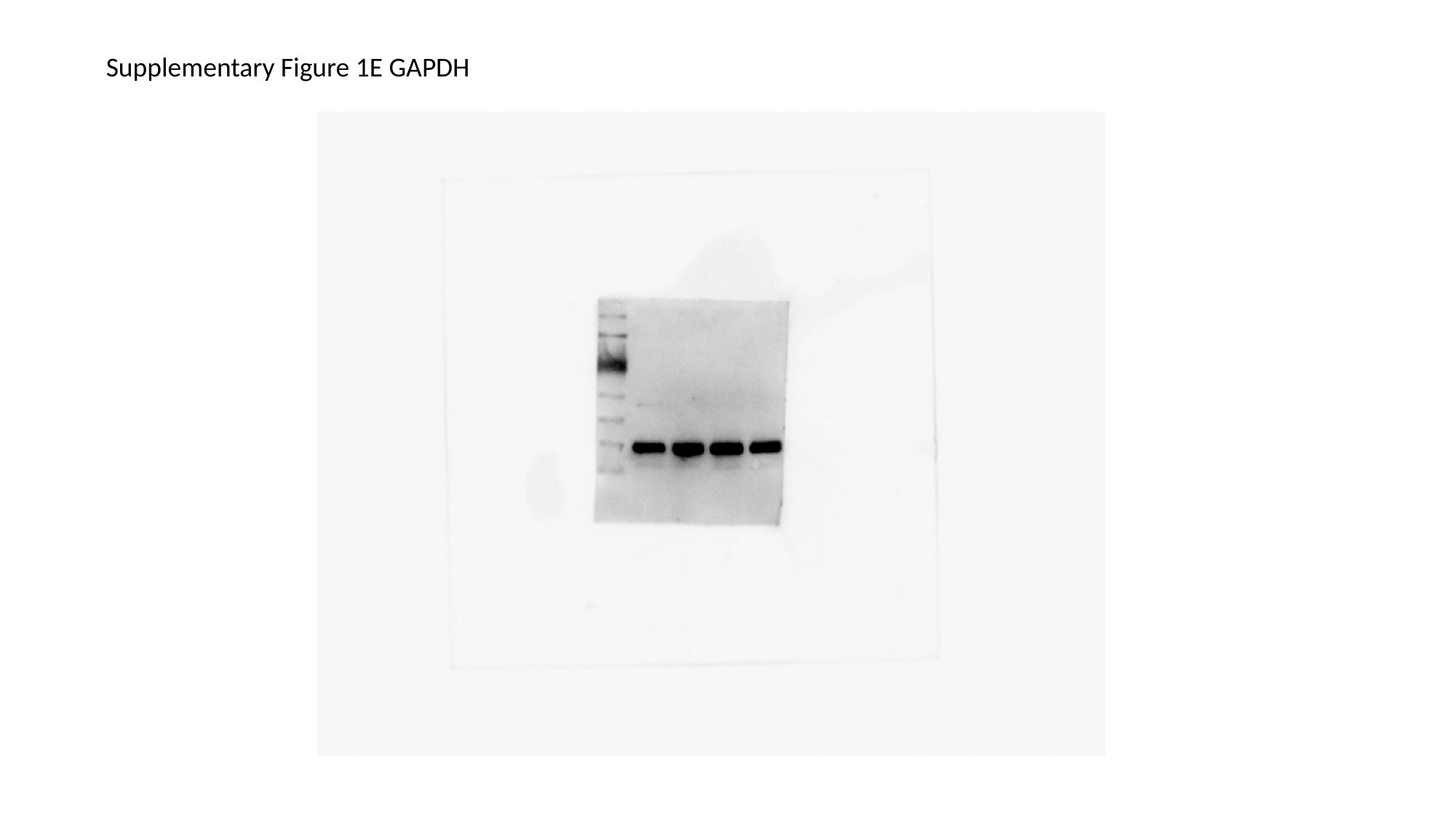

Supplementary Figure 1E GAPDH

## Slide 15
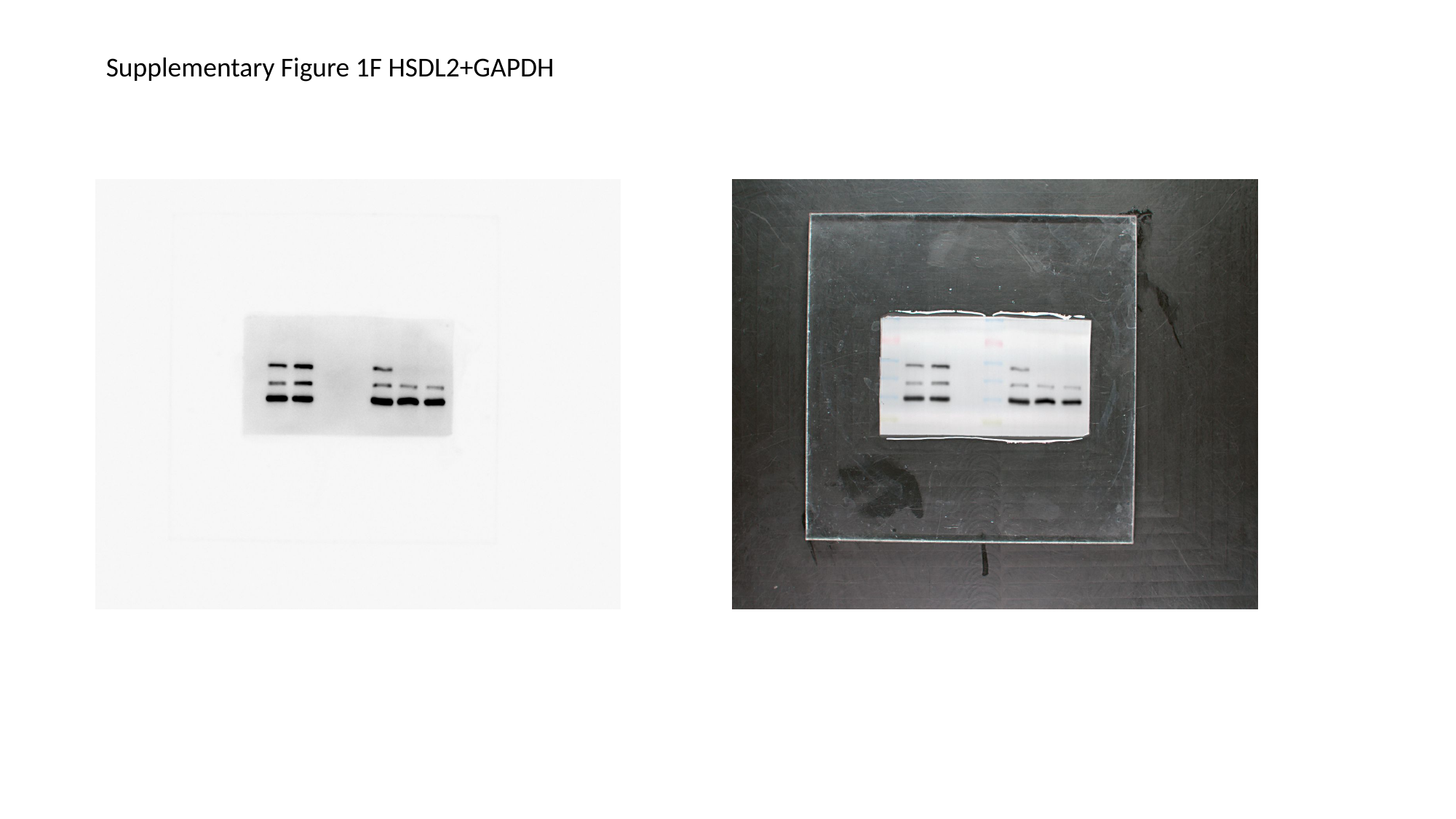

Supplementary Figure 1F HSDL2+GAPDH

## Slide 16
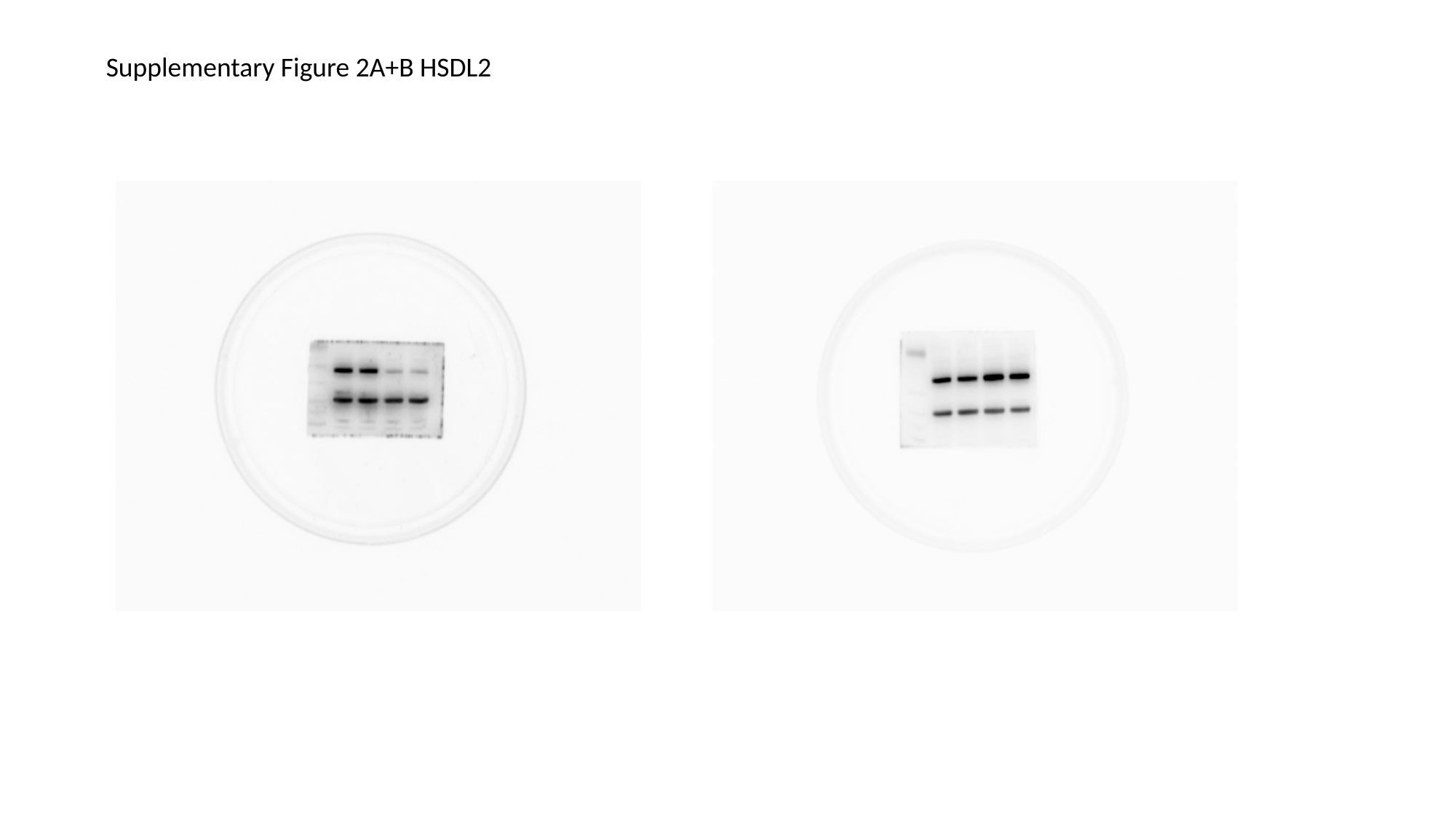

Supplementary Figure 2A+B HSDL2

## Slide 17
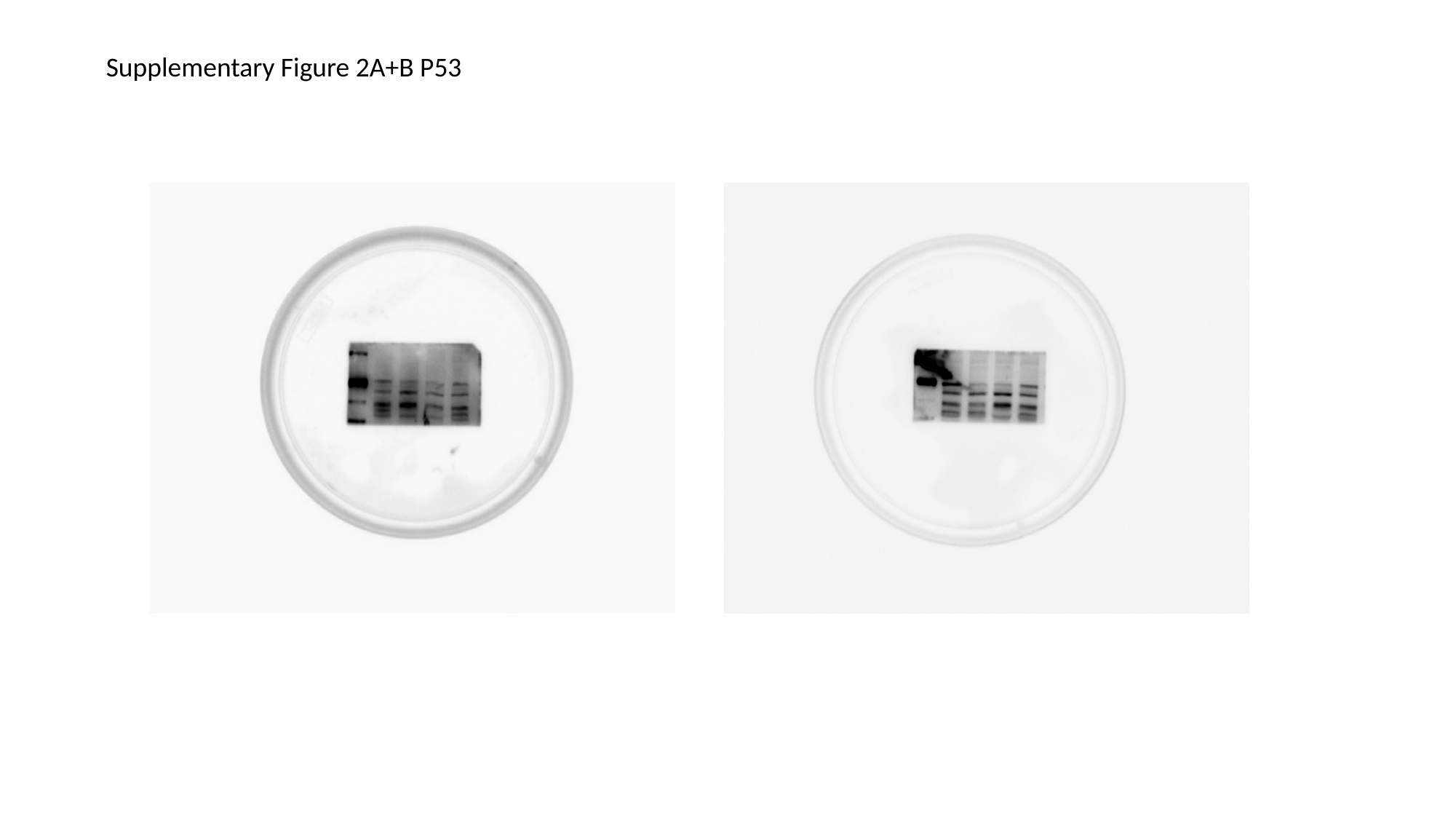

Supplementary Figure 2A+B P53

## Slide 18
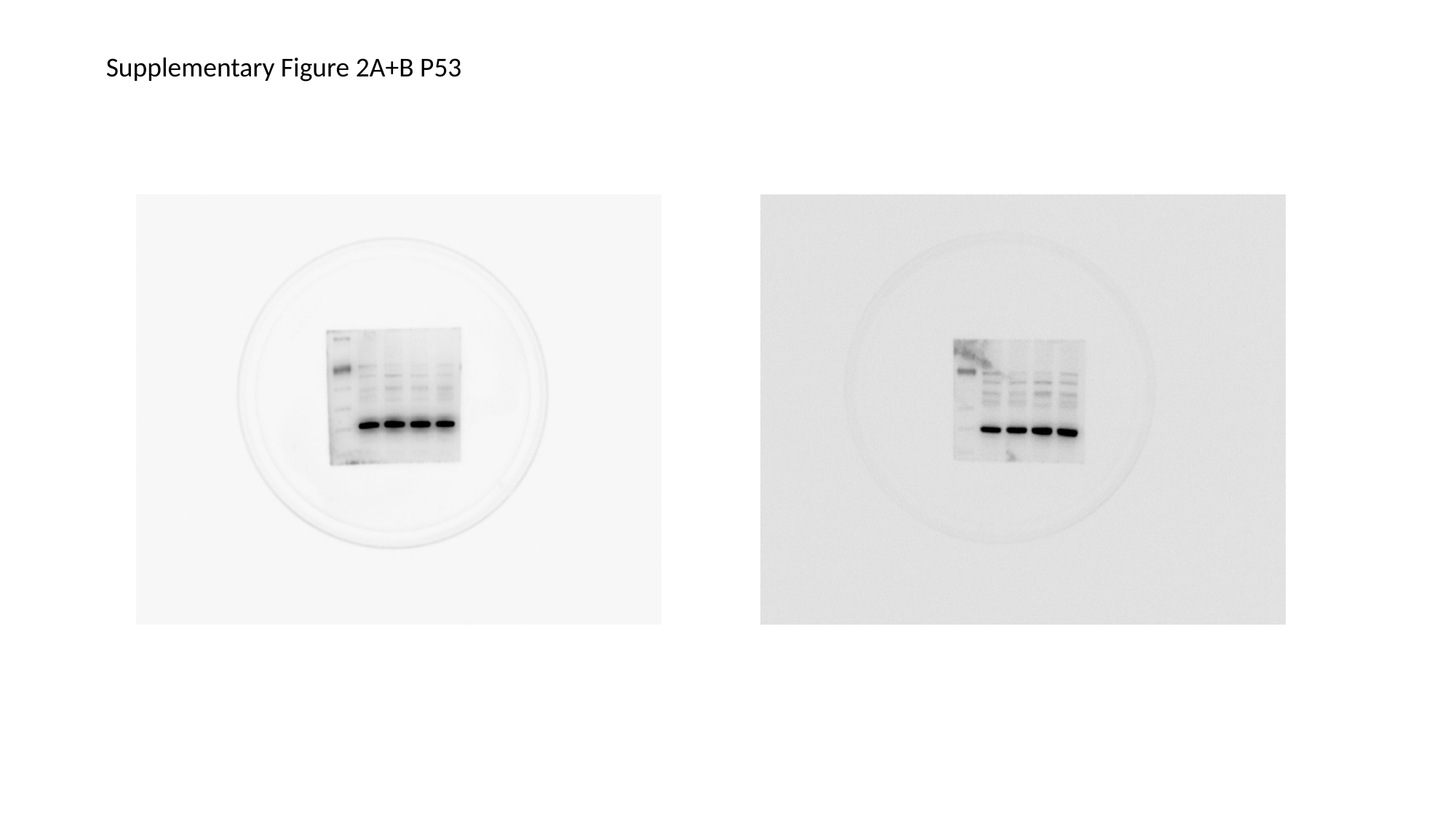

Supplementary Figure 2A+B P53

## Slide 19
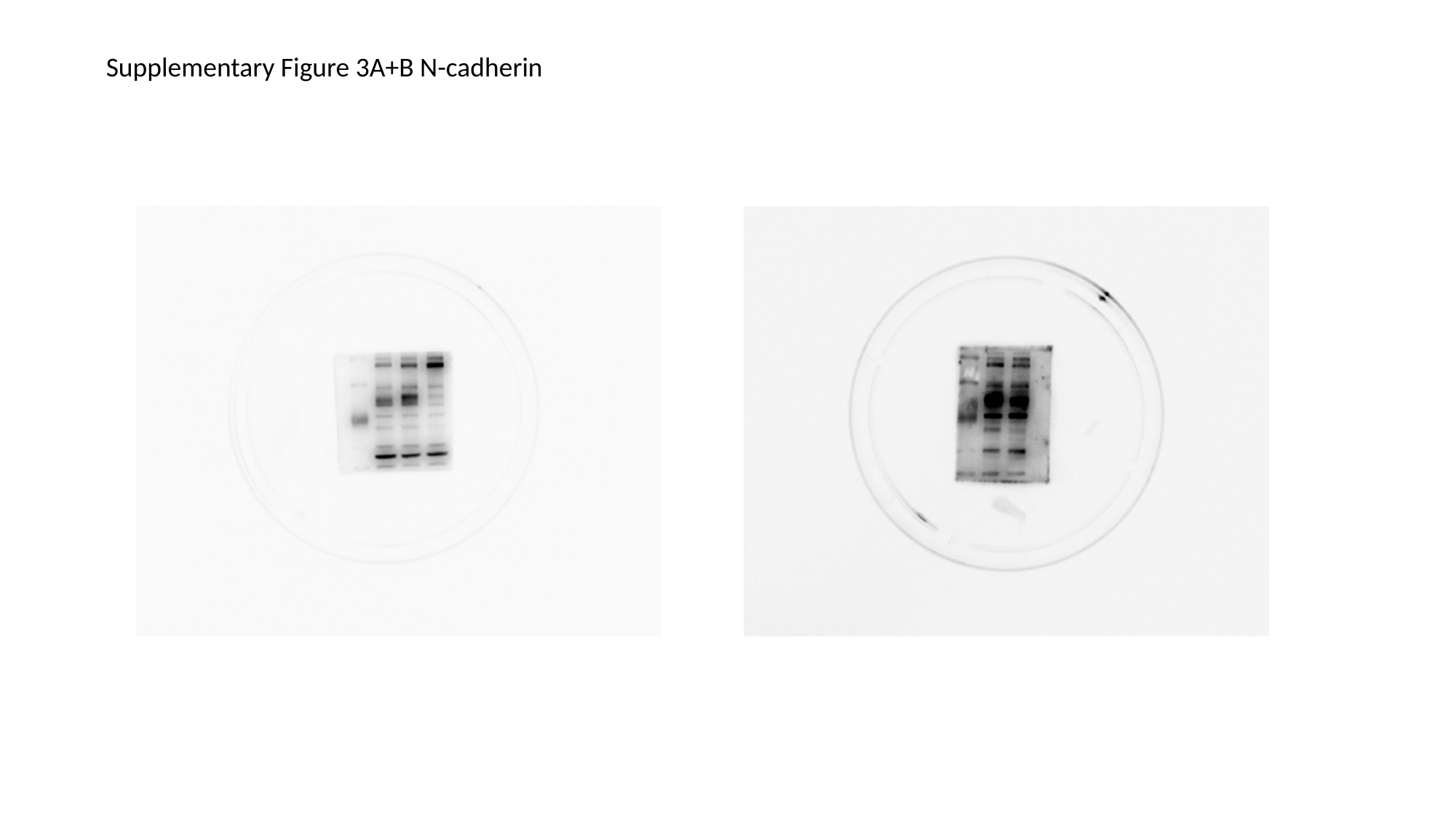

Supplementary Figure 3A+B N-cadherin

## Slide 20
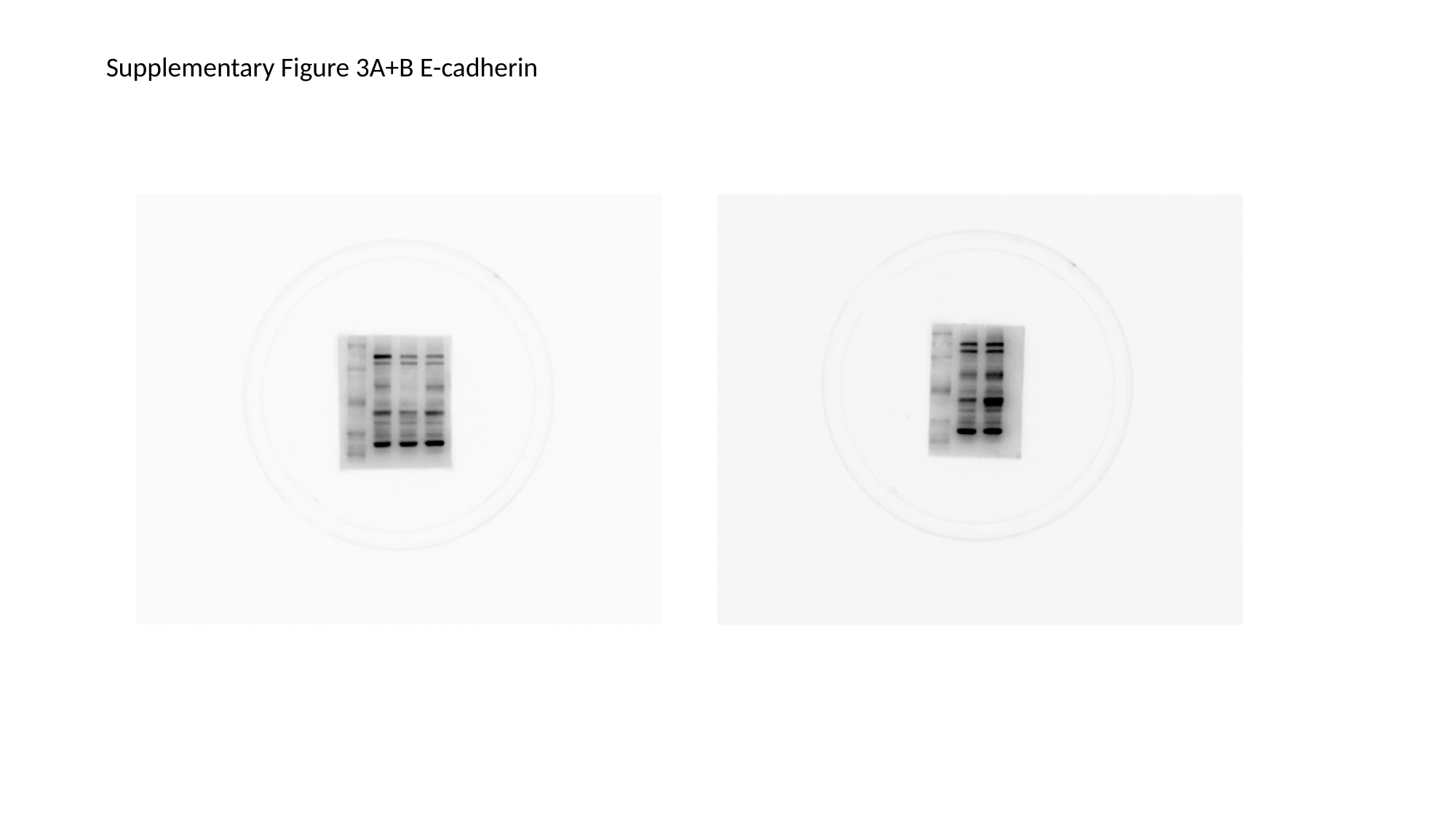

Supplementary Figure 3A+B E-cadherin

## Slide 21
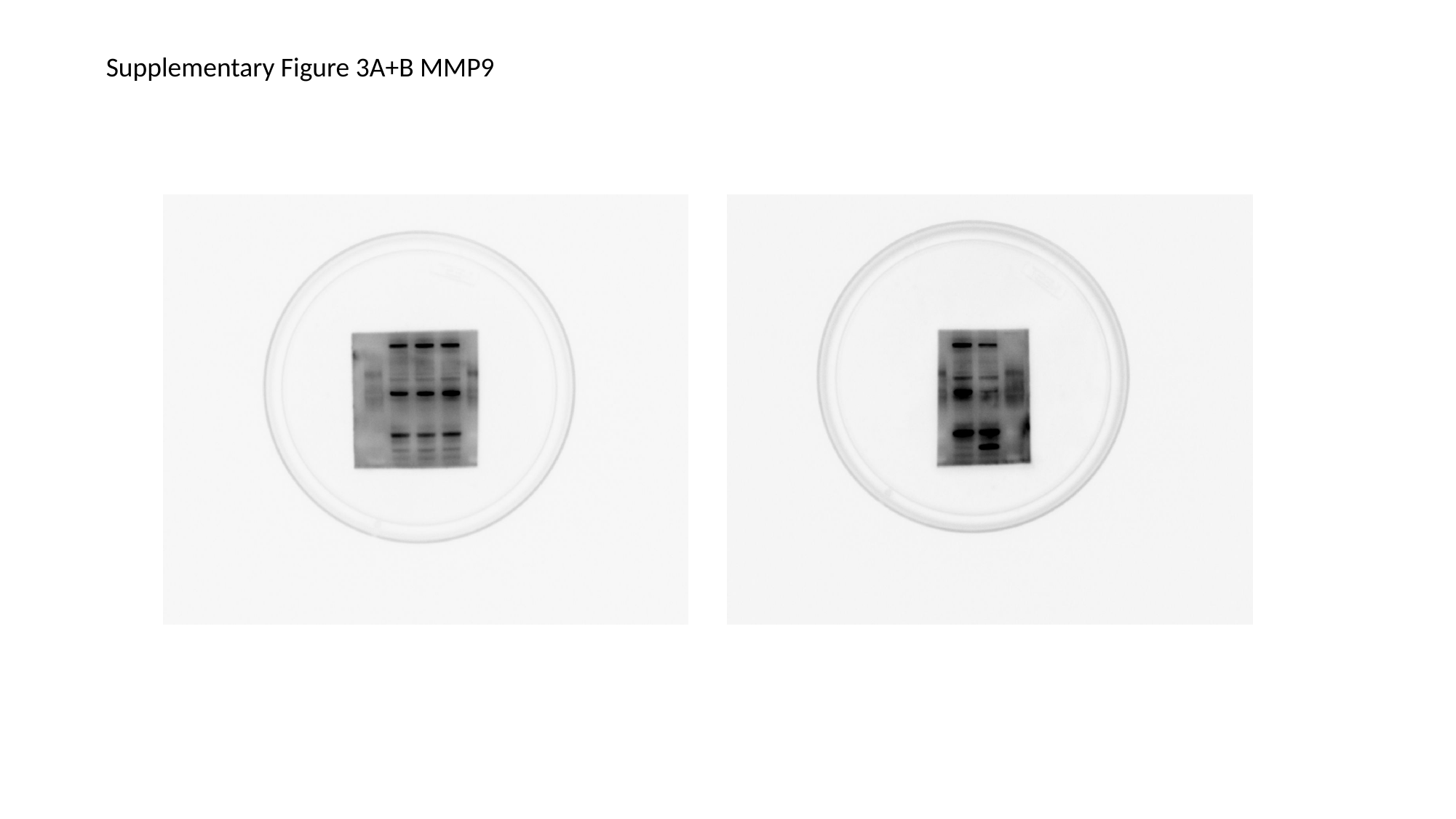

Supplementary Figure 3A+B MMP9

## Slide 22
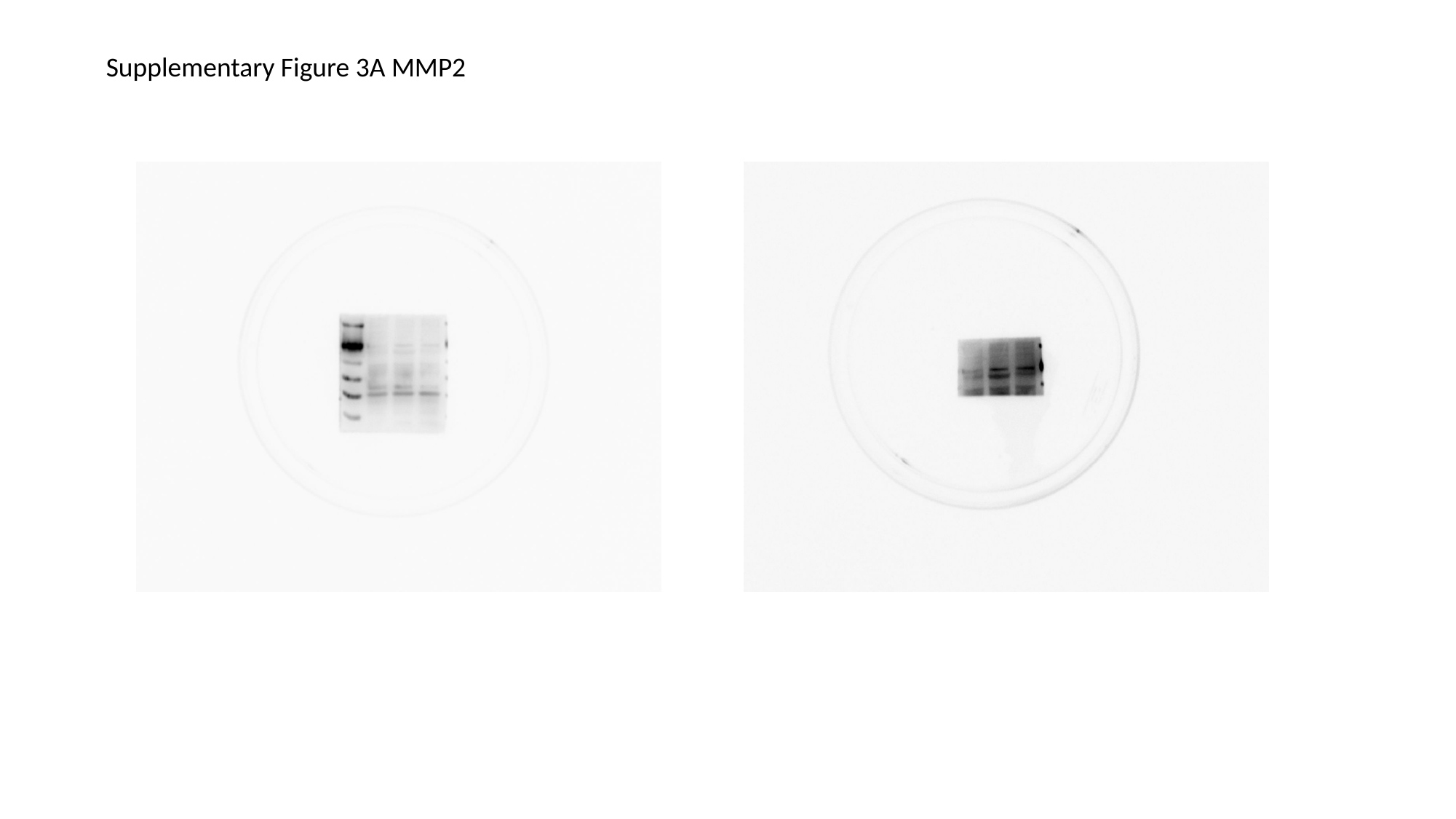

Supplementary Figure 3A MMP2

## Slide 23
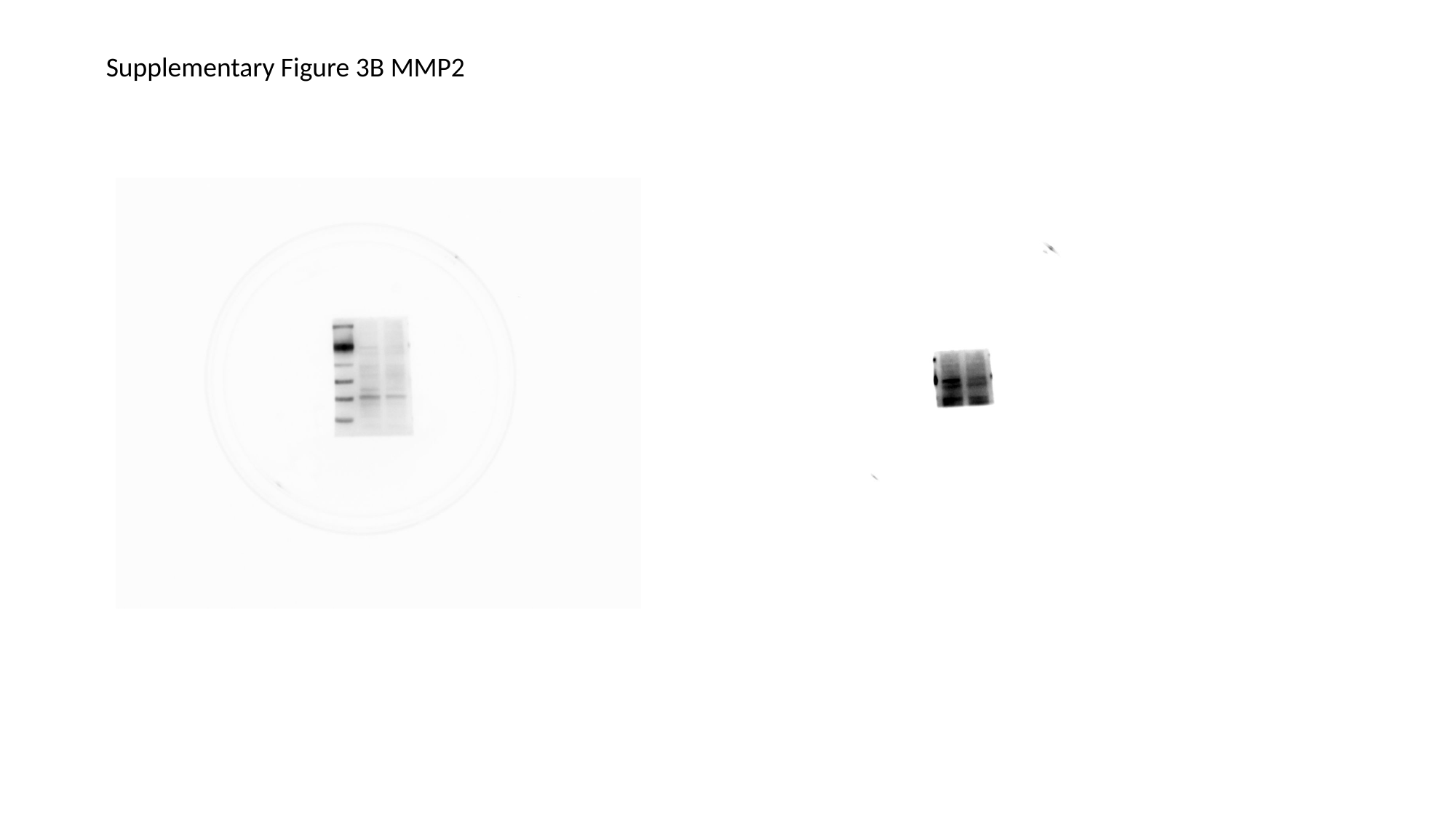

Supplementary Figure 3B MMP2

## Slide 24
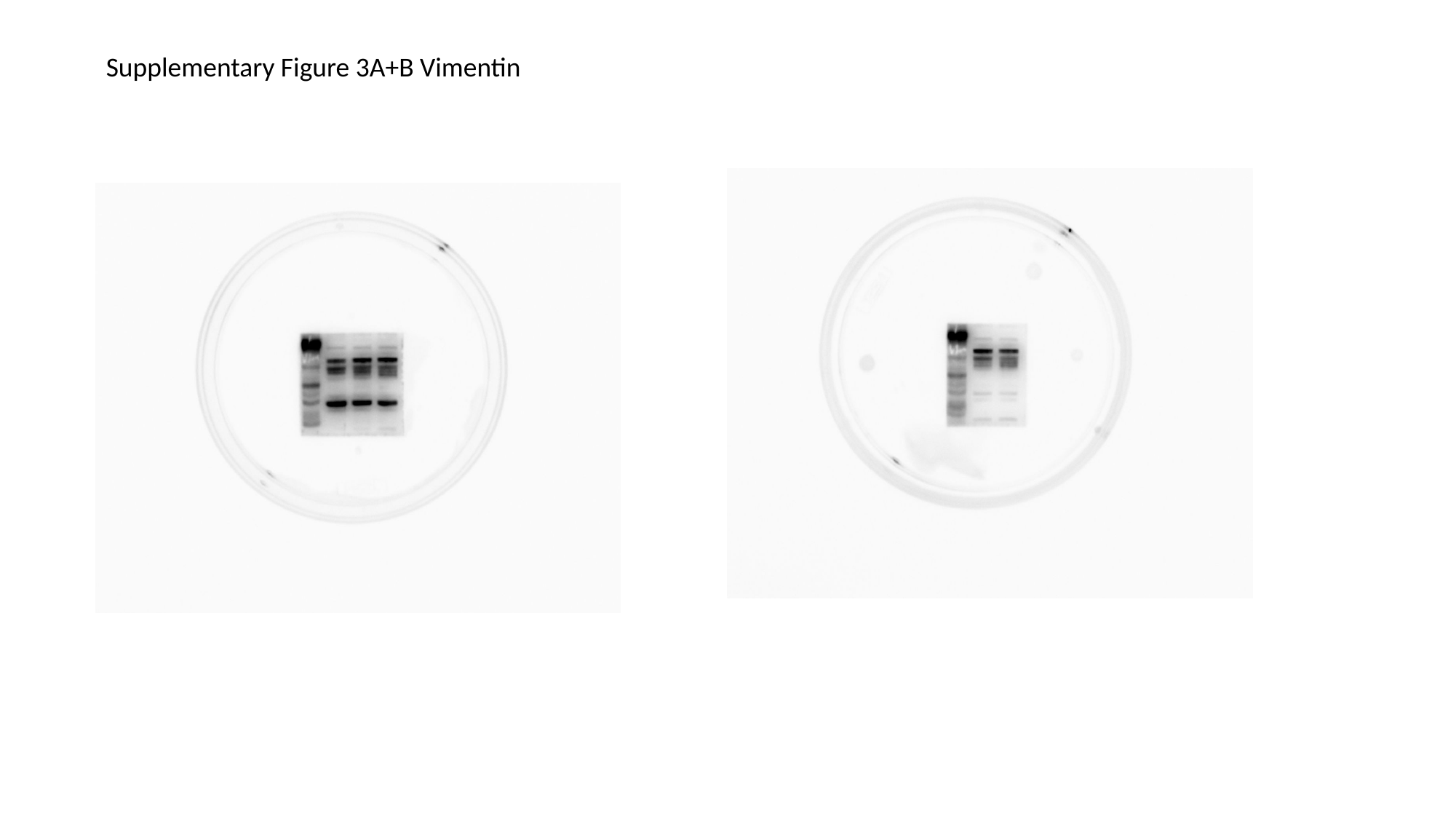

Supplementary Figure 3A+B Vimentin

## Slide 25
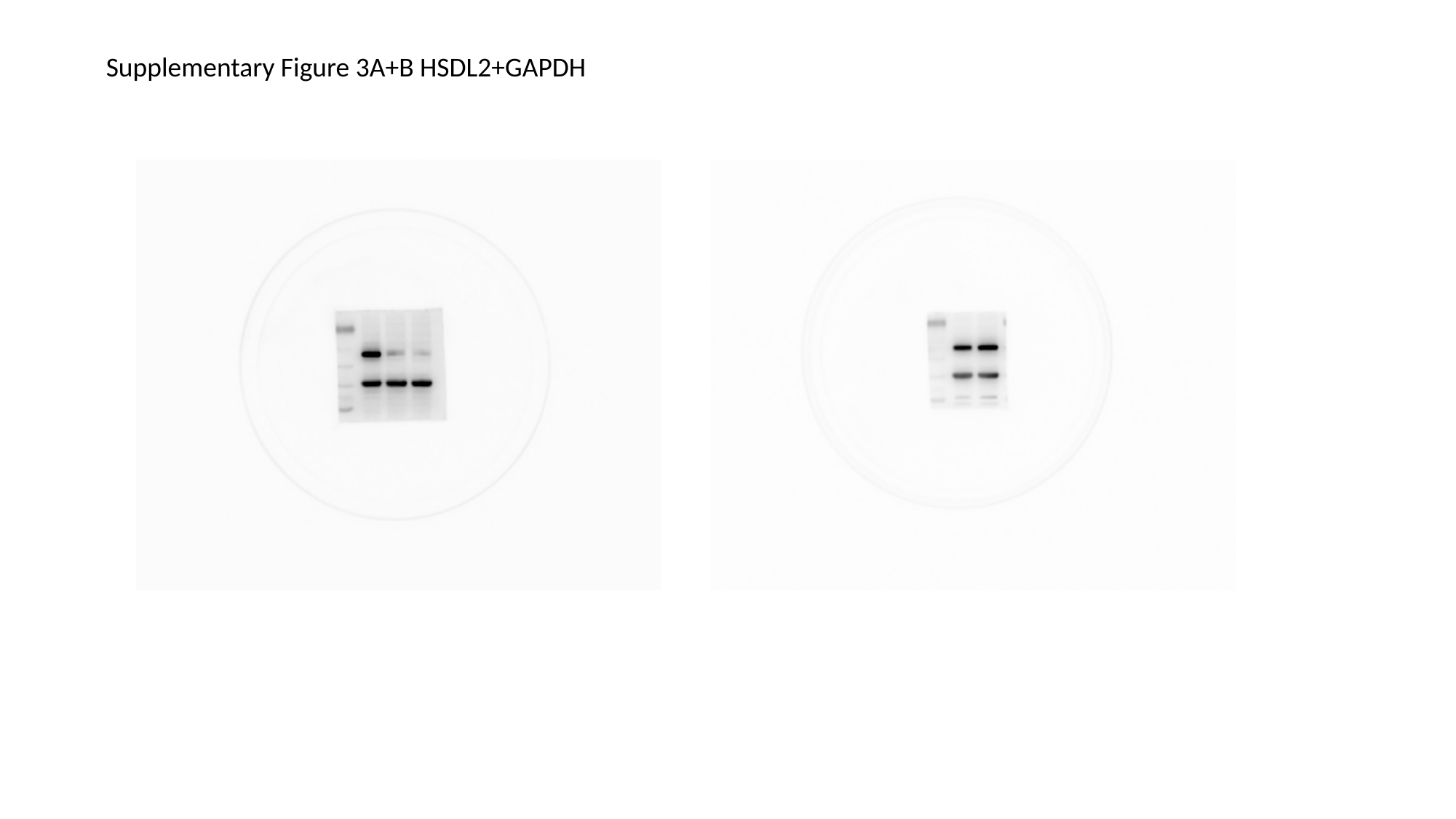

Supplementary Figure 3A+B HSDL2+GAPDH

## Slide 26
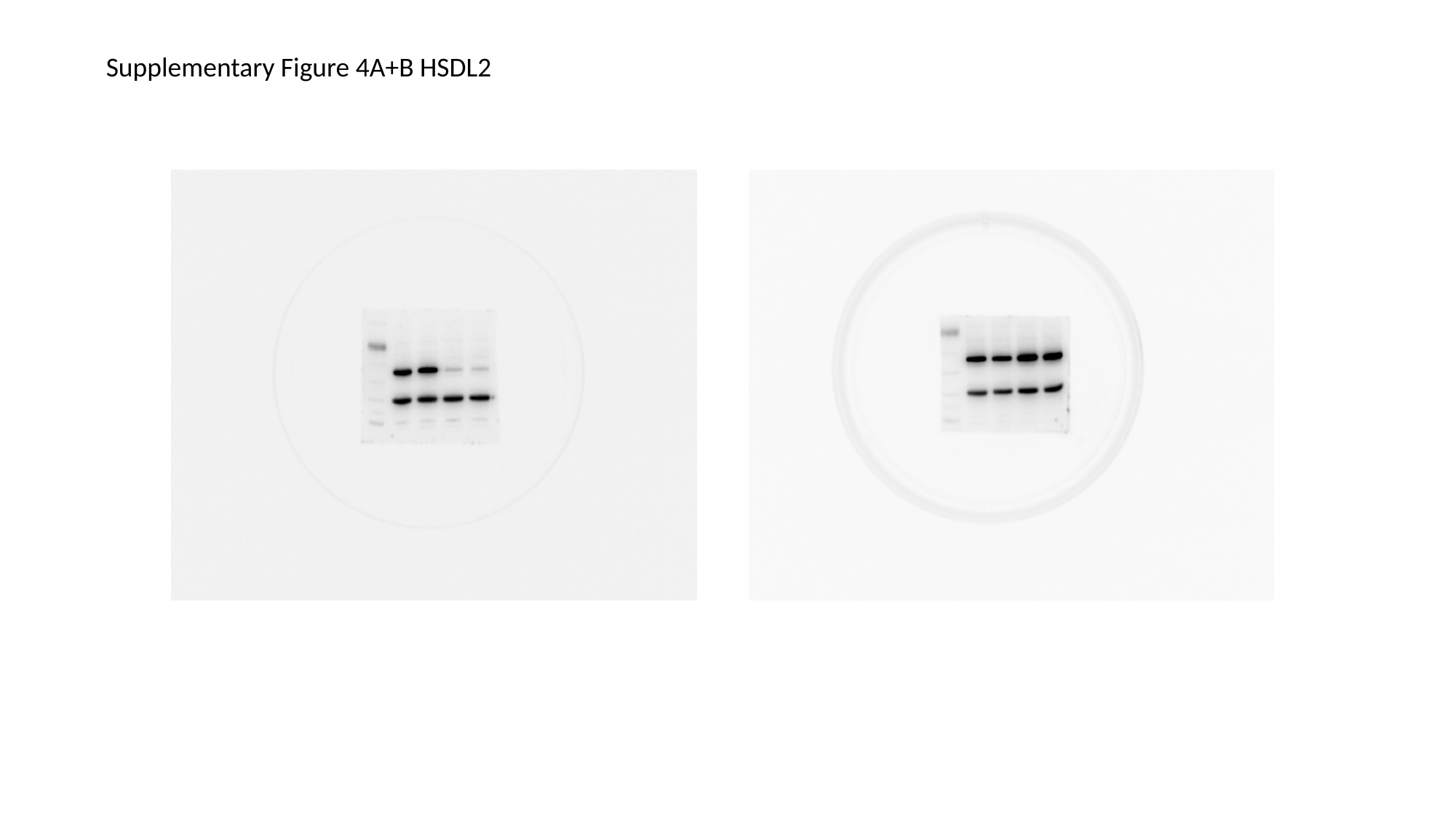

Supplementary Figure 4A+B HSDL2

## Slide 27
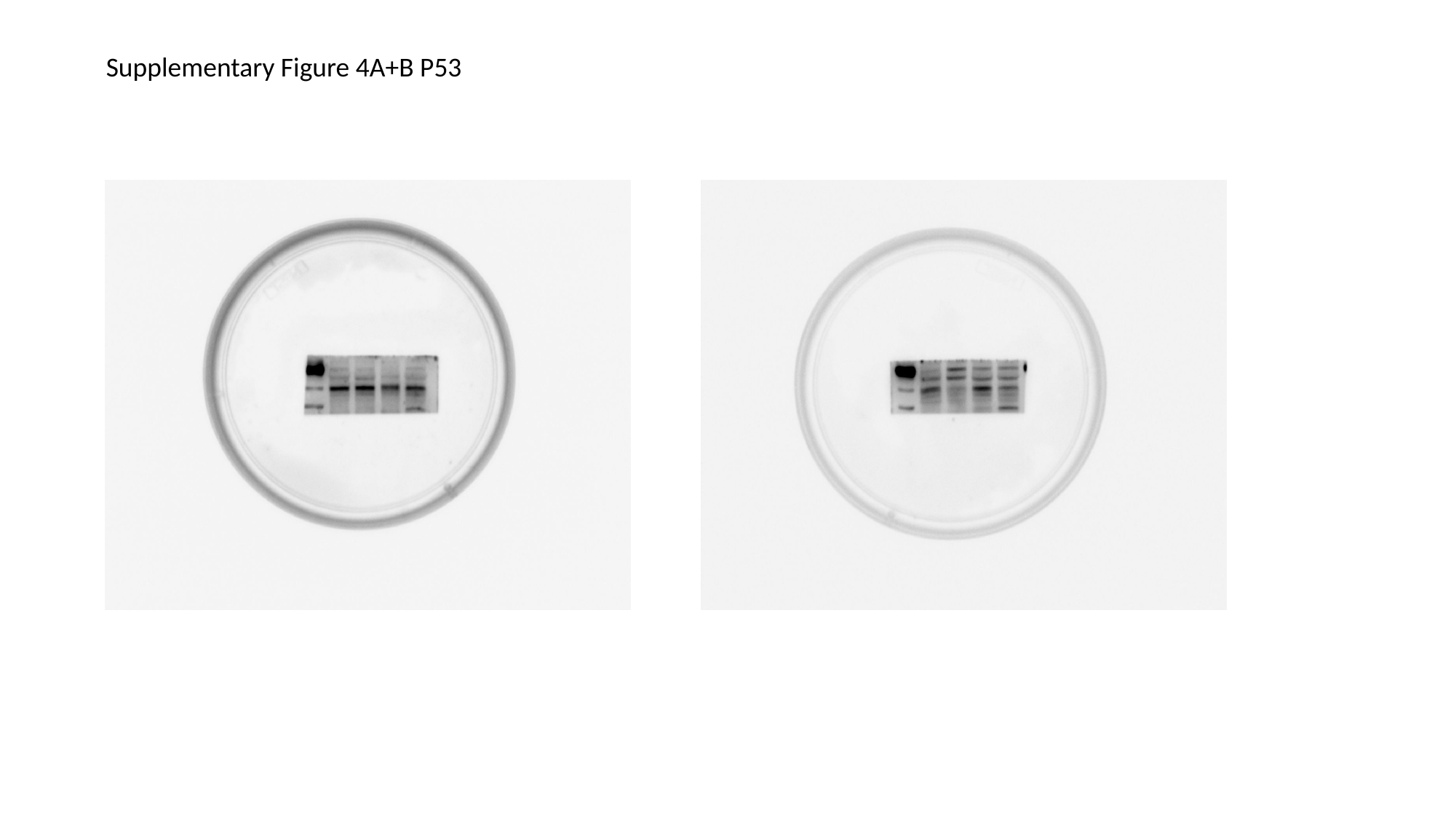

Supplementary Figure 4A+B P53

## Slide 28
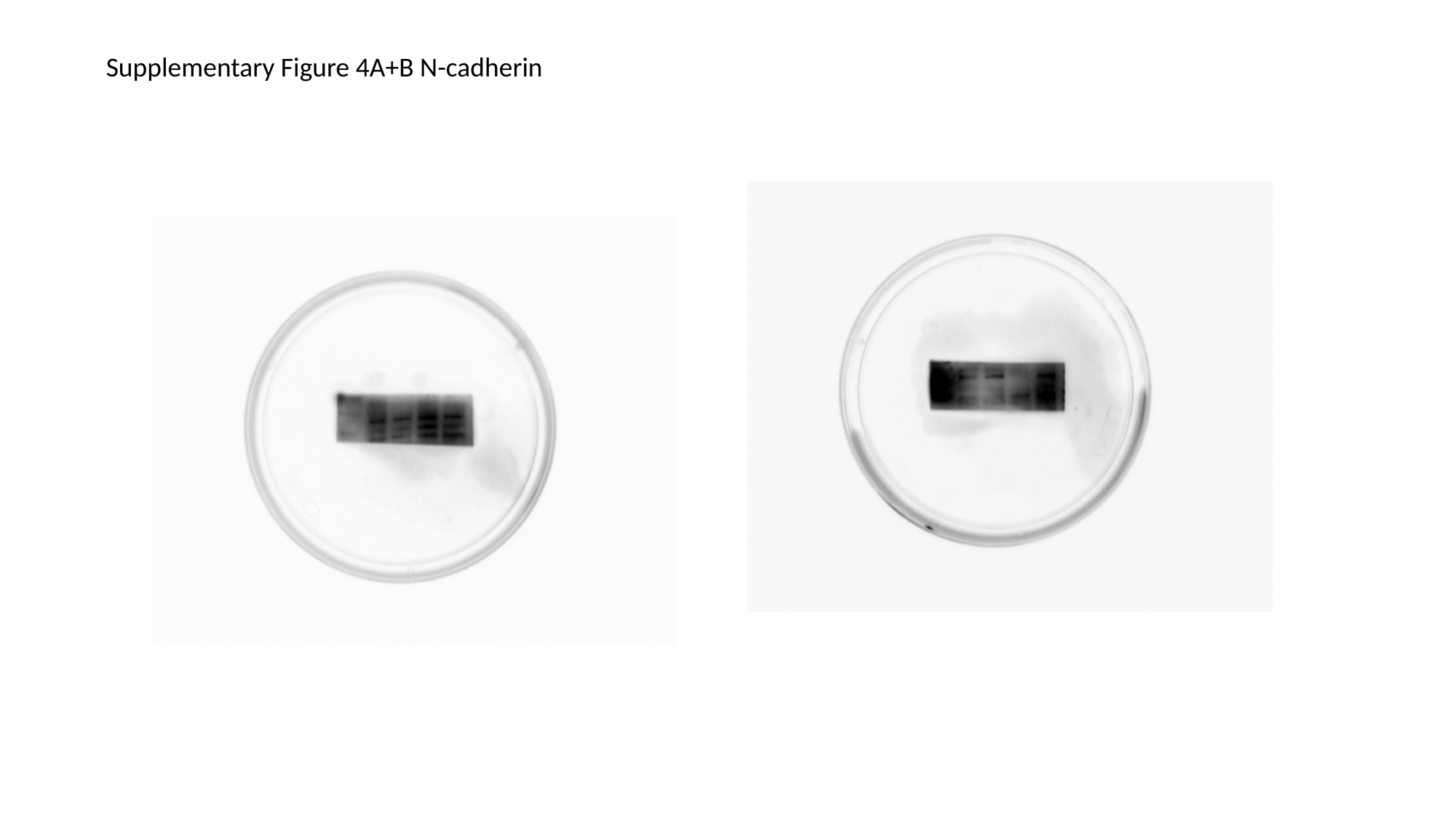

Supplementary Figure 4A+B N-cadherin

## Slide 29
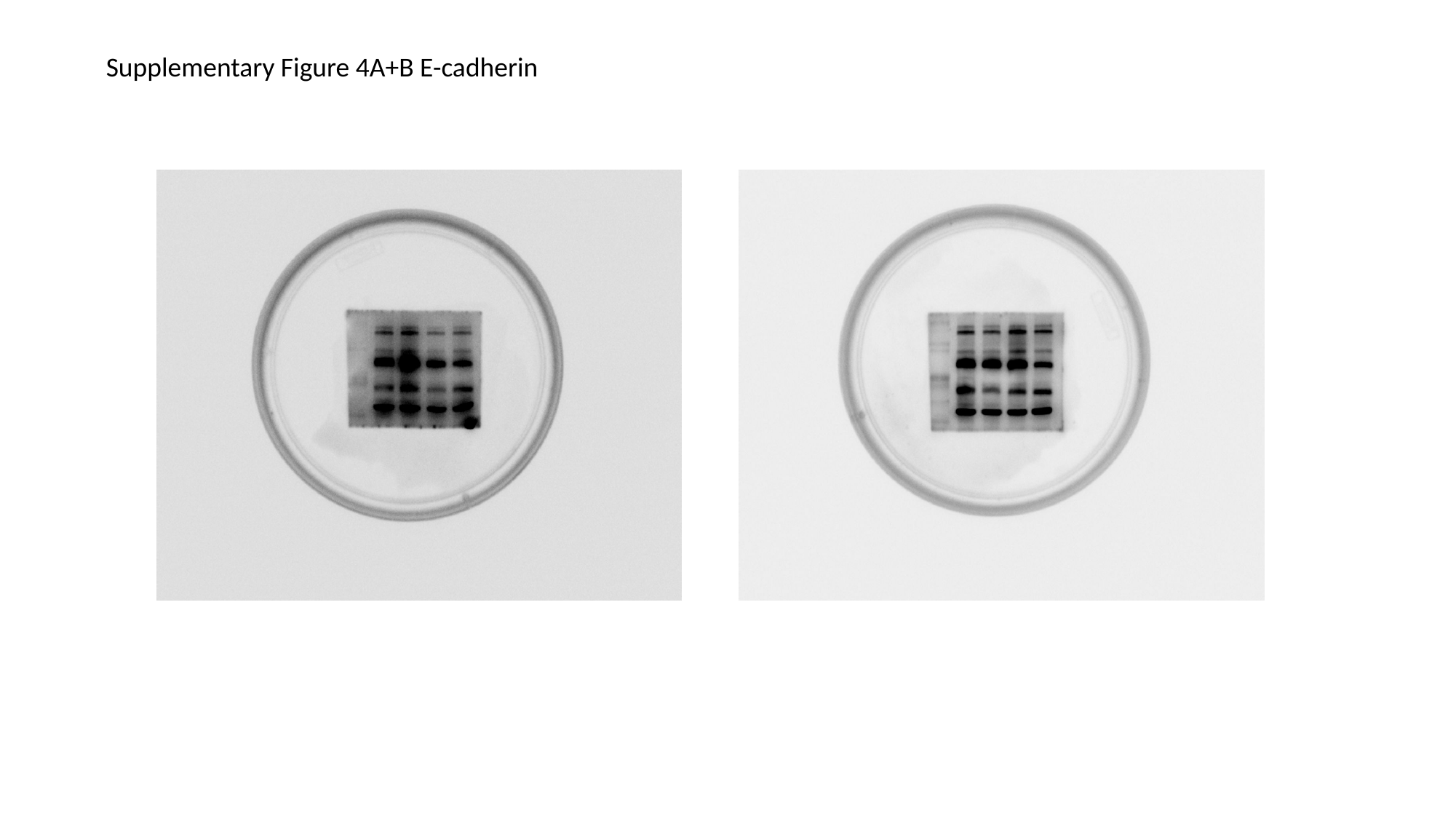

Supplementary Figure 4A+B E-cadherin

## Slide 30
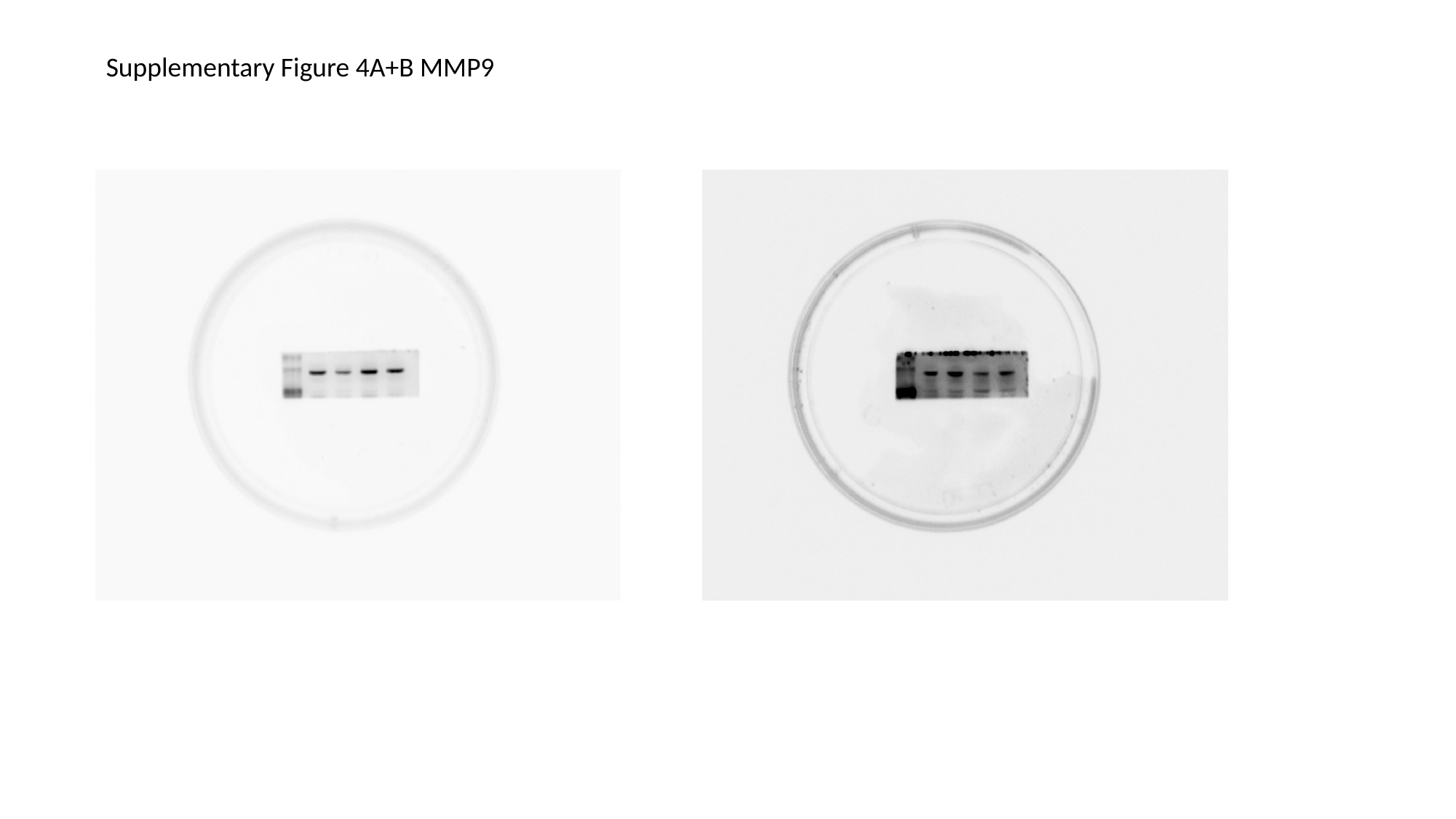

Supplementary Figure 4A+B MMP9

## Slide 31
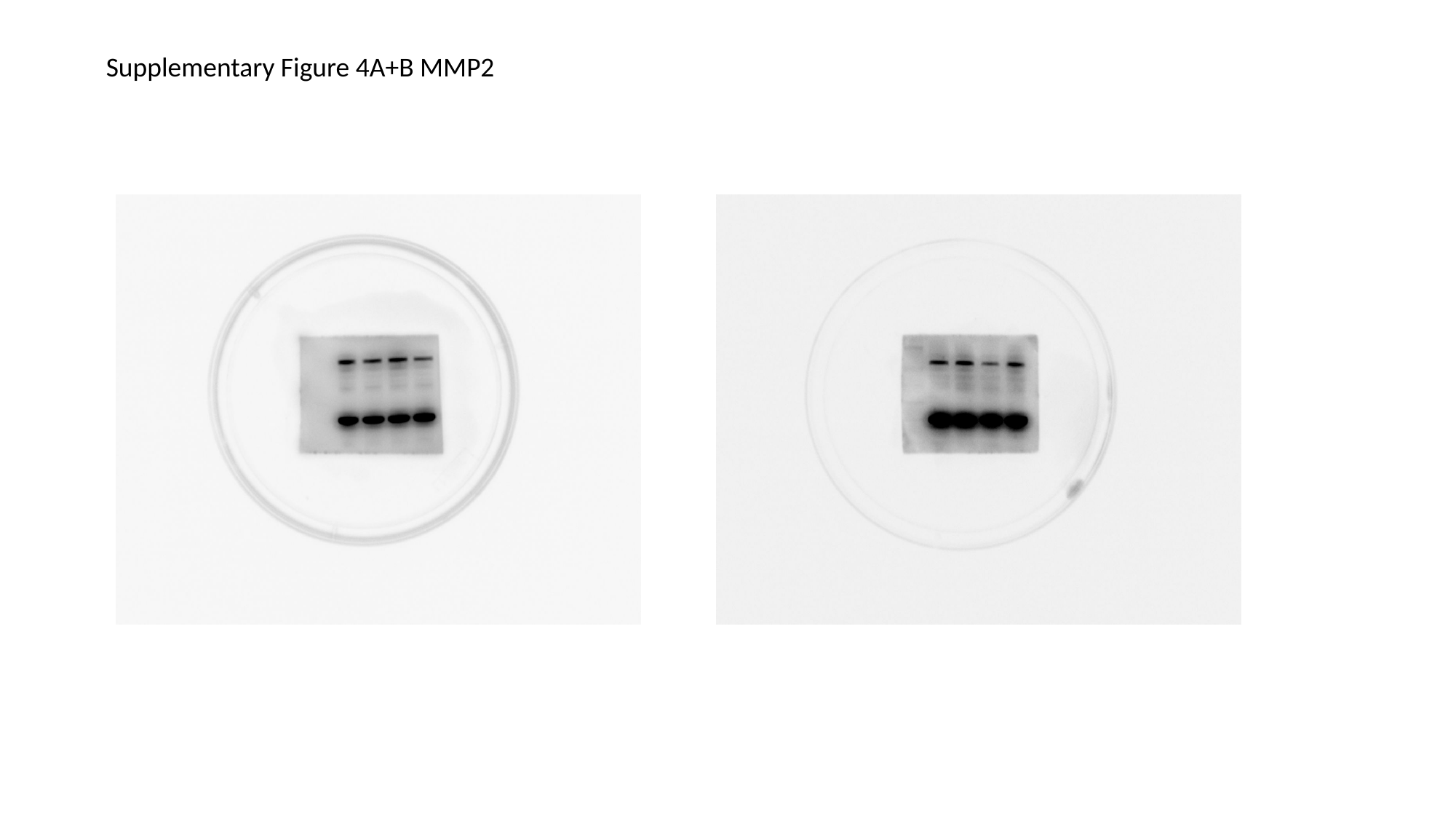

Supplementary Figure 4A+B MMP2

## Slide 32
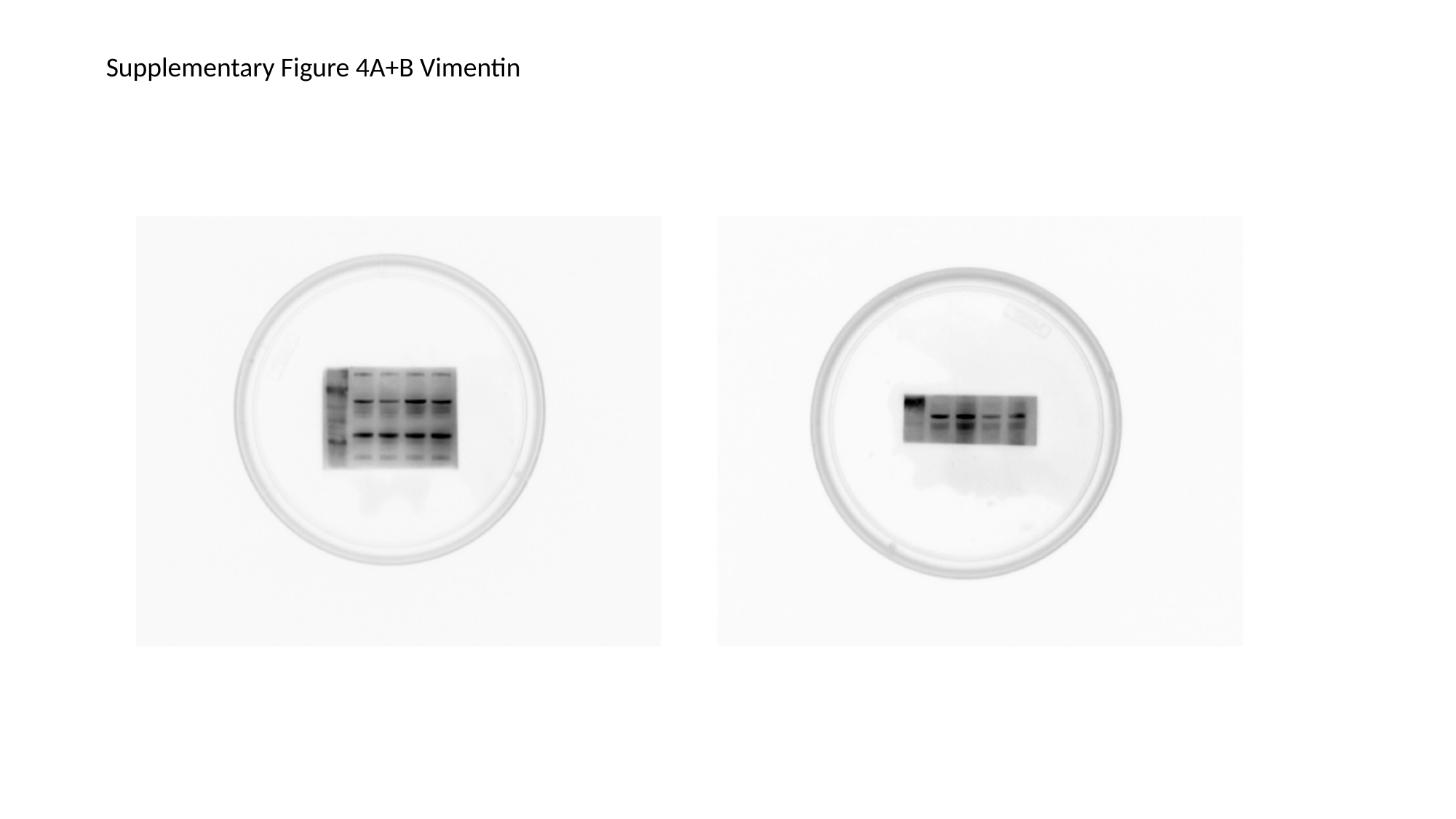

Supplementary Figure 4A+B Vimentin

## Slide 33
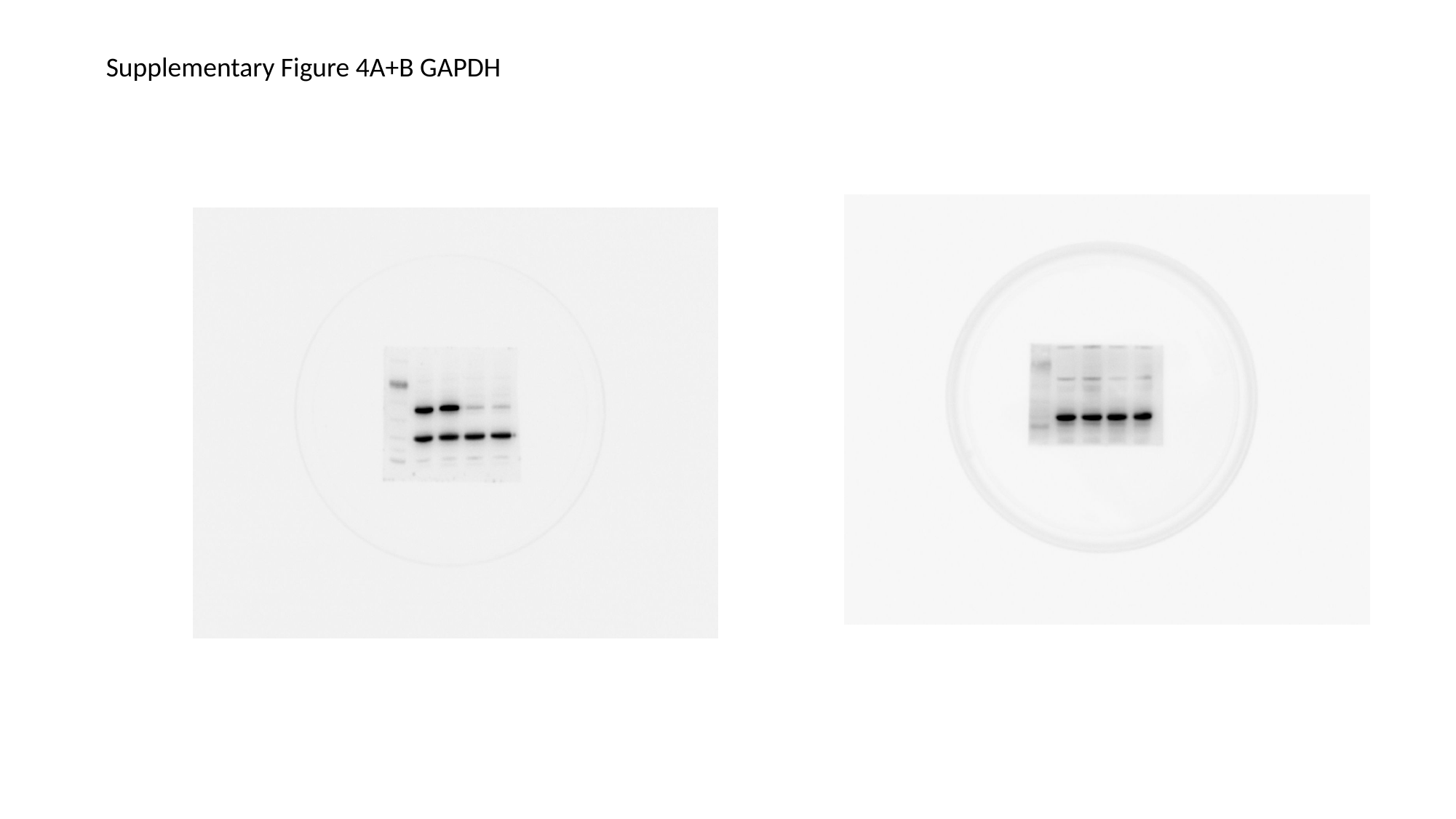

Supplementary Figure 4A+B GAPDH
